# Chromosome doubling to overcome the chrysanthemum cross barrier based on insight from transcriptomic and proteomic analyses

**DOI:** 10.1186/s12864-016-2939-0

**Published:** 2016-08-09

**Authors:** Fengjiao Zhang, Lichun Hua, Jiangsong Fei, Fan Wang, Yuan Liao, Weimin Fang, Fadi Chen, Nianjun Teng

**Affiliations:** 1 College of Horticulture, Nanjing Agricultural University, Nanjing, 210095 China; 2 Jiangsu Province Engineering Lab for Modern Facility Agriculture Technology and Equipment, Nanjing, 210095 China

**Keywords:** Chromosome doubling, *Chrysanthemum morifolium*, Cross barrier, Interspecific hybridization, iTRAQ, RNA-Seq

## Abstract

**Background:**

Cross breeding is the most commonly used method in chrysanthemum (*Chrysanthemum morifolium*) breeding; however, cross barriers always exist in these combinations. Many studies have shown that paternal chromosome doubling can often overcome hybridization barriers during cross breeding, although the underlying mechanism has seldom been investigated.

**Results:**

In this study, we performed two crosses: *C. morifolium* (pollen receptor) × diploid *C. nankingense* (pollen donor) and *C. morifolium* × tetraploid *C. nankingense.* Seeds were obtained only from the latter cross*.* RNA-Seq and isobaric tags for relative and absolute quantitation (iTRAQ) were used to investigate differentially expressed genes and proteins during key embryo development stages in the latter cross. A previously performed cross, *C. morifolium* × diploid *C. nankingense*, was compared to our results and revealed that transcription factors (i.e., the agamous-like MADS-box protein AGL80 and the leucine-rich repeat receptor protein kinase EXS), hormone-responsive genes (auxin-binding protein 1), genes and proteins related to metabolism (ATP-citrate synthase, citrate synthase and malate dehydrogenase) and other genes reported to contribute to embryo development (i.e., *LEA*, elongation factor and tubulin) had higher expression levels in the *C. morifolium* × tetraploid *C. nankingense* cross. In contrast, genes related to senescence and cell death were down-regulated in the *C. morifolium* × tetraploid *C. nankingense* cross.

**Conclusions:**

The data resources helped elucidate the gene and protein expression profiles and identify functional genes during different development stages. When the chromosomes from the male parent are doubled, the genes contributing to normal embryo developmentare more abundant. However, genes with negative functions were suppressed, suggesting that chromosome doubling may epigenetically inhibit the expression of these genes and allow the embryo to develop normally.

**Electronic supplementary material:**

The online version of this article (doi:10.1186/s12864-016-2939-0) contains supplementary material, which is available to authorized users.

## Background

Intergeneric/interspecific crosses between cultivars and their wild species are widely used to improve the tolerance of crop plants to biotic and abiotic stresses. The offspring of these crosses might have greater environmental adaptability and species diversity. The embryos develop normally, and seeds can be successfully obtained, which are the considerations when evaluating crossing efficiency. Many factors influence the efficiency of interspecific crosses. Differences in the ploidy levels of the parental plants were thought to be one major barrier causing endosperm malformation and the inhibition of germination [1]. Indeed, hybrid embryos are often spontaneously aborted due to the absence of endosperm or retarded development after successful interspecific pollination, which is a typical post-fertilization barrier that strongly hampers embryo development [2]. There is growing evidence that polyploid breeding has the potential to overcome this barrier and has emerged as one of the most efficient methods. The development of the major crop species common wheat (*Triticum aestivum*) is a universally acknowledged textbook example of an allohexaploid derived through hybridization between a domesticated forms of the tetraploid *T. turgidum* ssp. *dicoccoides* and the diploid *Aegilops tauschii* [3, 4]. In 1988, Badger found that tetraploid azaleas could overcome interspecific barriers. The cross *Rhododendron calendulaceum* x tetraploid evergreen Obtusum was highly successful, and produced many viable seeds. However, the cross between *R. calendulaceum* and the diploid evergreen Obtusum was seldom successful [5]. Although chromosome doubling can also overcome reproductive barriers during the cross breeding of many other plant species, the underlying mechanism remains unknown.

*C. morifolium* is an important ornamental crop that is similar to the rose and lily with a high demand in the consumer market. To meet increasing consumer needs, breeders must search for novel traits, improve plant qualities and increase the resistance of existing species to biotic or abiotic stresses. Cross breeding is the conventional path to improve genetic variability and develop modified species. Successful intergeneric hybridization efforts have been reported between the chrysanthemum and related genera, such as *C. lavandulifolium* × *Ajania remotipinna*, *A. remotipinna* × *C. chanetii* [6] and *Opisthopappus taihangensis* × *C. lavandulifolium* [7]. However, interspecific crosses with the chrysanthemum are difficult because of its limited genetic diversity, especially between the hexaploid chrysanthemum and diploid wild species. In a cross between *C. morifolium* and diploid *C. nankingense*, six interspecific hybrids were generated but required ovary rescue [8]. In a cross chrysanthemum cultivar ‘Zhongshanzixing’ × diploid *C. nankingense*, no seeds were obtained, whereas seeds were obtained in the cross between ‘Zhongshanzixing’ and tetraploid *C. nankingense* [9]. These results suggest that polyploidization of the male parent can overcome cross barriers between incongruous groups. In these studies, the reason for interspecific cross inhibition was attentively examined. A pre- or post-fertilization barrier was confirmed to exist between different hybrids in a large number of morphological and cytological studies [8, 10]. However, the expression patterns of genes and proteins underlying the morphological and cytological traits remain unclear. The effects of polyploidization on interspecific chrysanthemum crosses have not been studied, and the mechanisms by which different ploidy levels regulate chrysanthemum embryonic development remain elusive.

Currently, RNA-Seq is often used for gene discovery and transcript abundance measurements during a certain state in particular organs or tissues and has been successfully applied in different species [11]. Recently, transcriptome and proteome methodologies were applied to developing *Medicago truncatula* seeds [12] and *Arabidopsis* seed germination [13]. Deep sequencing analysis was applied to the peanut pod transcriptome to identify candidate genes related to early embryo abortion [14]; this approach was also used to study the early *Arabidopsis* embryo [15]. In a previous study, we explored genes and proteins associated with chrysanthemum embryo abortion in the cross *C. morifolium* × diploid *C. nankingense* using RNA-Seq and isobaric tags for relative and absolute quantitation (iTRAQ), focusing on the perspective of embryo abortion [16]. Here, we performed the cross *C. morifolium* × tetraploid *C. nankingense*, which decreased the embryo abortion rate and produced seeds, but the genes and proteins that aided normal embryo development were unclear. Therefore, the combination of transcriptomic and proteomic data is suitable to study chrysanthemum embryo development and explore the differential expression patterns in different interspecific crosses and samples. Our objectives were as follows: (i) to analyze gene and protein expression in the developing chrysanthemum embryo and characterize expression changes at different stages of embryonic development from the cross *C. morifolium* × tetraploid *C. nankingense* and to identify over-expressed genes and proteins that promote normal embryo development; (ii) to compare the expression of key genes and proteins related to chrysanthemum embryo abortion in the two different crosses using a previous study [16] and point out the genes and proteins with positive roles promoting chrysanthemum embryo development. This study will also provide a better understanding of the mechanism underlying the different seed setting rates when the male parent has different ploidy levels.

## Results

### Ovule development and seed setting

**Fig. 1:**
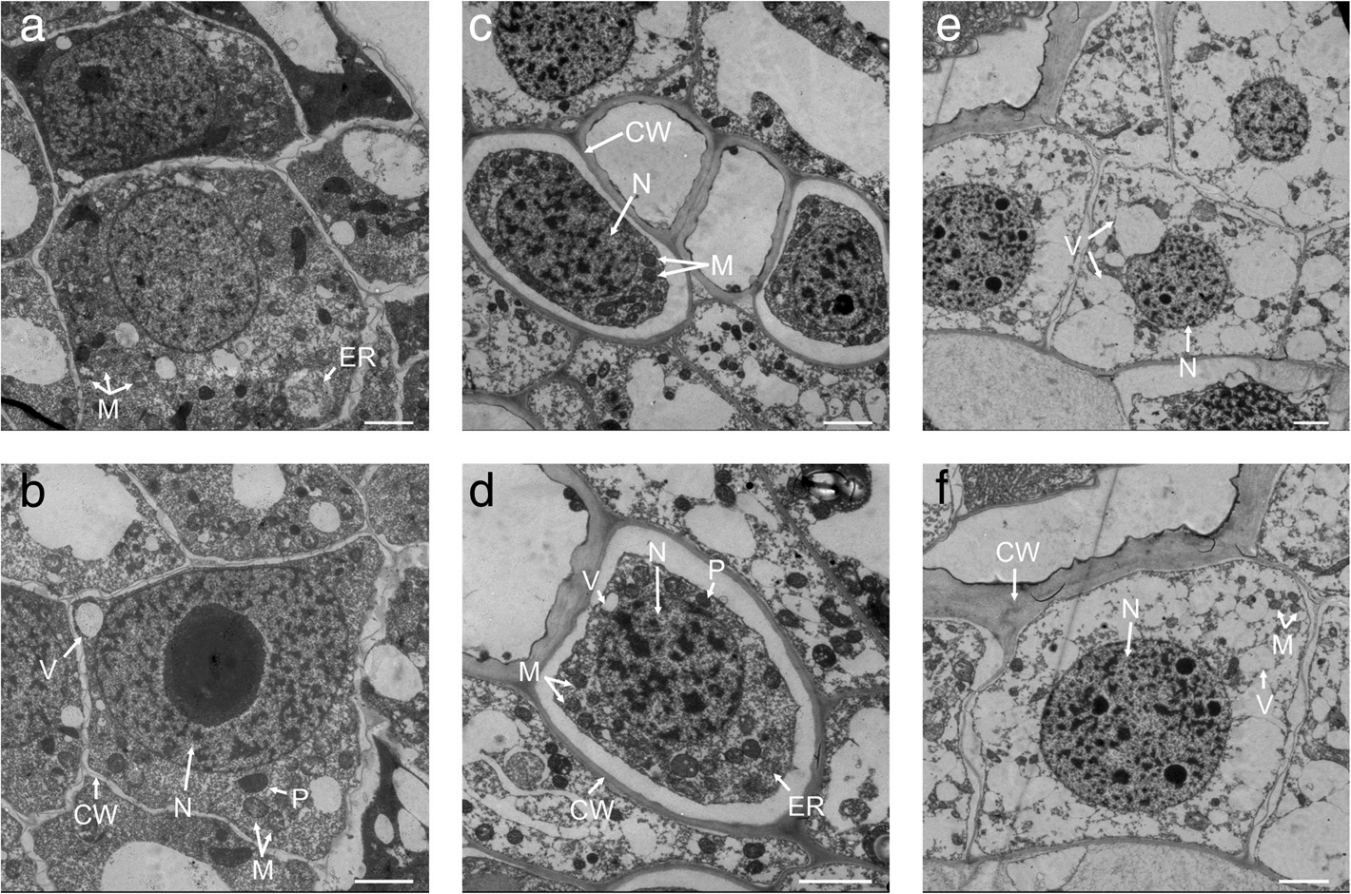
TEM of NE12, NE18 and AE18 in the cross *C. morifolium* × tetraploid *C. nankingense*. **a**, **b** NE12; **c**, **d** NE18; **e**, **f** AE18. Bar = 20 μm. NE12: normal embryos at 12 days after pollination; NE18: normal embryos at 18 days after pollination; AE18: abnormal embryos at 18 days after pollination. N: nucleus; CW: cell wall; M: mitochondria; V: vacuole; P: plastid; ER: endoplasmic reticulum

In the cross *C. morifolium* × tetraploid *C. nankingense*, many ovules were morphologically well developed with a full form at 12 days after pollination (DAP); a total of 93.5 ± 1.7 % of the ovules were normal. The percentage was 89.7 ± 3.1 % in the cross *C. morifolium* × diploid *C. nankingense*, similar to that of the *C. morifolium* × tetraploid *C. nankingense* cross. Based on continuous observations, many ovules were shriveled, and only 54.8 ± 0.9 % of the ovules appeared normal at 18 DAP; however, this value was significantly higher than 43.3 ± 1.8 %, the corresponding rate in the *C. morifolium* × diploid *C. nankingense* cross. Eventually, a few seeds were obtained generating a seed set rate of 1.45 ± 0.03 % in this study. No seeds were obtained when the diploid *C. nankingense* was the male parent [16] (Table 1). In this study, transmission electron microscopy (TEM) analysis showed that the normal cells at 12 DAP contained many organelles, mitochondria and plastids with normal shapes. Moreover, the cells exhibited an intact cell wall structure and rich edge information. In the normal embryos at 18 DAP, although the cytoplasm contracted slightly as the embryos developed, the organelles were well developed and metabolism was robust in the mitochondria. However, abnormal embryos at 18 DAP showed significant differences, including clearly shrinking nuclei and a decrease in mitochondria and plastid. Additionally, the organelle structures were aberrant, with degradation and obvious variation and thickening of the cell wall (Fig. 1).

**Table 1:** Percentage of normal embryos at different stages after pollination in *C. morifolium* × *C. nankingense*

Cross	Percentage of normal embryos or seed set		
	12 DAP	18 DAP	40 DAP
*C. morifolium* × tetraploid *C. nankingense*	93.5 ± 1.7 %	54.8 ± 0.9 %	1.45 ± 0.03 %
*C. morifolium* × diploid *C. nankingense* [16]	89.7 ± 3.1 %	43.3 ± 1.8 %^*^	0^*^

DAP means days after pollination.

‘*’ indicates the significant differences at *P* ≤ 0.05 according to *t*-test. Values are means ± standard error (*n* = 3).

### Transcriptome sequencing and read assembly

Using Illumina high-throughput sequencing, we constructed three cDNA libraries from normal embryos at 12 DAP (NE12), normal embryos at 18 DAP (NE18) and abnormal embryo at 18 DAP (AE18) from the *C. morifolium* × tetraploid *C. nankingense* cross. After cleaning the raw data, we obtained 52,206,996, 51,935,854 and 51,720,046 clean reads from NE12, NE18 and AE18, respectively, containing 4,698,629,640, 4,674,226,860 and 4,654,804,140 clean nucleotides (Table 2). Based on these high-quality clean reads, a total of 99,119 unigenes were assembled with a mean length of 726 nt, including 45,770 clusters and 53,349 singletons. The numbers of unigenes for NE12, NE18 and AE18 were 88,909, 91,971 and 97,889, respectively, with a mean length of 550-580 nt (Table 3).

**Table 2:** Output statistics of sequencing

Sample	Total raw reads	Total clean reads	Total clean nucleotides (nt)	Q20 percentage	N percentage	GC percentage
NE12	55,087,918	52,206,996	4,698,629,640	98.37 %	0.00 %	44.20 %
NE18	56,564,364	51,935,854	4,674,226,860	98.40 %	0.00 %	44.49 %
AE18	58,160,684	51,720,046	4,654,804,140	98.45 %	0.00 %	44.19 %

**Table 3:** Statistics of assembly quality

Sample	Total number of sequences	Total Length (nt)	Mean Length (nt)	Distinct Clusters	Distinct Singletons
NE12	88,909	51,281,289	577	33,867	55,042
NE18	91,971	52,587,899	572	34,581	57,390
AE18	97,889	54,412,745	556	36,097	61,792
All	99,119	71,965,184	726	45,770	53,349

### Unigene functional annotation

Annotation analysis of the chrysanthemum embryo provided information on gene expression and the function of all unigenes detected at different developmental stages. The annotation consisted of protein functional annotation, pathway annotation, Clusters of Orthologous Groups of proteins (COG) functional annotation and gene ontology (GO) functional annotation. As a result, 58,799 unigenes were annotated with the NR, NT, Swiss-Prot, KEGG, COG and GO databases; the numbers of annotations obtained from each database were 56,665, 39,101, 36,897, 33,594, 20,391 and 43,526, respectively (Table 4). The largest number of annotated unigenes was obtained from the NR database.

**Table 4:** Annotation of unigenes in chrysanthemum embryo

Sequence database	Number of annotated unigenes	Percentage of annotated unigenes
NR	56,665	96.37
NT	39,101	66.5
Swiss-Prot	36,897	62.75
KEGG	33,594	57.13
COG	20,391	34.68
GO	43,526	74.03
All	58,799	100

To investigate unigene function and evaluate the effectiveness of the annotation process, 20,391 sequences accounting for 34.68 % of the total annotated unigenes were assigned a COG functional annotation. Among the 25 COG categories, the clusters in the top three were ‘general function prediction only’ (6749); ‘transcription’ (3844); and ‘replication, and recombination and repair’ (3323). The number of unigenes in each cluster accounted for more than 15 % of all unigenes with a COG annotation. The two clusters with the fewest unigenes were ‘extracellular structures’ (14) and ‘nuclear structure’ (11); the percentage of unigenes in each category was less than 1 % (Additional file 1: Figure S1).

Using NR annotation, we obtained GO functional annotations describing the properties of genes and their products in chrysanthemum. Based on sequence homology, 43,526 sequences could be categorized into 55 functional groups, including the three main GO classifications (biological process, cellular component, and molecular function). There were 24, 15 and 16 functional subcategories in each main classification; ‘cellular process’, ‘cell’ and ‘cell part’ (equal), and ‘catalytic activity’ were the most common subcategories (Additional file 2: Figure S2). Additionally, the classes ‘organelle’ and ‘metabolic process’ contained many unigenes that might play important roles in the metabolic pathways involved in chrysanthemum embryo development.

Next, the KEGG pathway database was applied to identify the biological pathways activated in the chrysanthemum embryo. A total of 33,594 annotated unigenes were assigned to 128 KEGG pathways (Additional file 3: Table S1). The majority of these pathways were ‘metabolic pathways’ (22.67 %), ‘biosynthesis of secondary metabolites’ (11.27 %), ‘plant-pathogen interaction’ (5.67 %) and ‘plant hormone signal transduction’ (4.97 %).

### Genes related to embryo development and differentially expressed genes at three stages

**Fig. 2:**
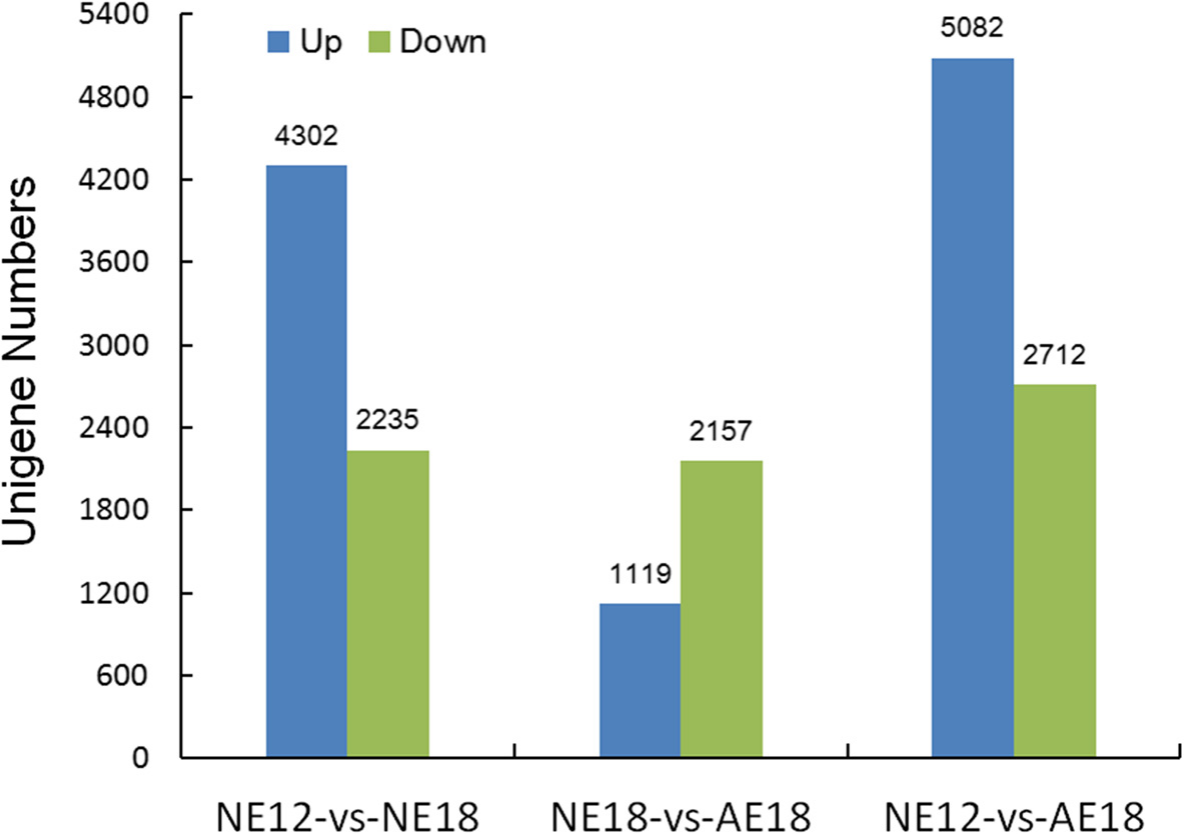
Differentially expressed genes among three libraries in the cross *C. morifolium* × tetraploid *C. nankingense*

Using FPKM (Fragments Per kb per Million fragments), we explored the gene expression levels in normal and abnormal embryos. In three comparisons (NE12 vs NE18, NE18 vs AE18, and NE12 vs AE18), the number of differentially expressed genes (DEGs) was 6537 (4302 were up-regulated), 3276 (1119 were up-regulated), and 7794 (5082 were up-regulated). The first and third comparisons exhibited more up-regulated genes than down-regulated genes (Fig. 2). The details of the DEGs are presented in Additional file 4: Table S2. During the embryonic developmental stage from 12 DAP to 18 DAP (including both normal and abnormal embryos), the number of up-regulated genes at 18 DAP was nearly twice as high as the number of down-regulated genes. Interestingly, the number of down-regulated genes in abnormal embryos was higher than the number of up-regulated genes during the later developmental stage (18 DAP) (Fig. 2).

**Fig. 3:**
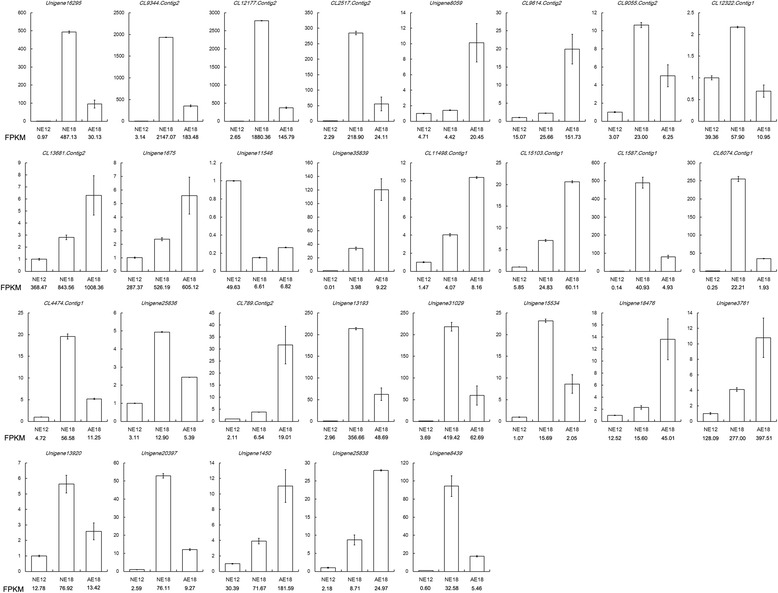
The expression patterns of DEGs in two transcriptome libraries. Blue columns represent the cross *C. morifolium*  × tetraploid *C. nankingense*, and green columns represent the cross* C. morifolium*  × diploid *C. nankingense*

After deep analysis of these DEGs, we found that several categories of genes had similar expression patterns in normal and abnormal embryos. Then, we used the CDS sequences of these DEGs as a query to identify the same unigenes in the transcriptome library sequenced from chrysanthemum embryos in the *C. morifolium* × diploid *C. nankingense* cross [16]. We analyzed the expression of some of the genes to obtain clues concerning their roles during chrysanthemum embryo development (i.e., *CmLEA*, *CmLEC*, *CmEM*, *CmSSP*, *CmOLE*, *CmTUB* and *CmEXT*). The expression patterns of these genes differed between the two crosses and various samples. For instance, *CmTUB* in NE18 had the highest expression level in the cross *C. morifolium* × tetraploid *C. nankingense*; however, in the cross *C. morifolium* × diploid *C. nankingense,* it was most highly expressed during NE12 (Fig. 3).

### Pathway classification by KEGG

**Fig. 4:**
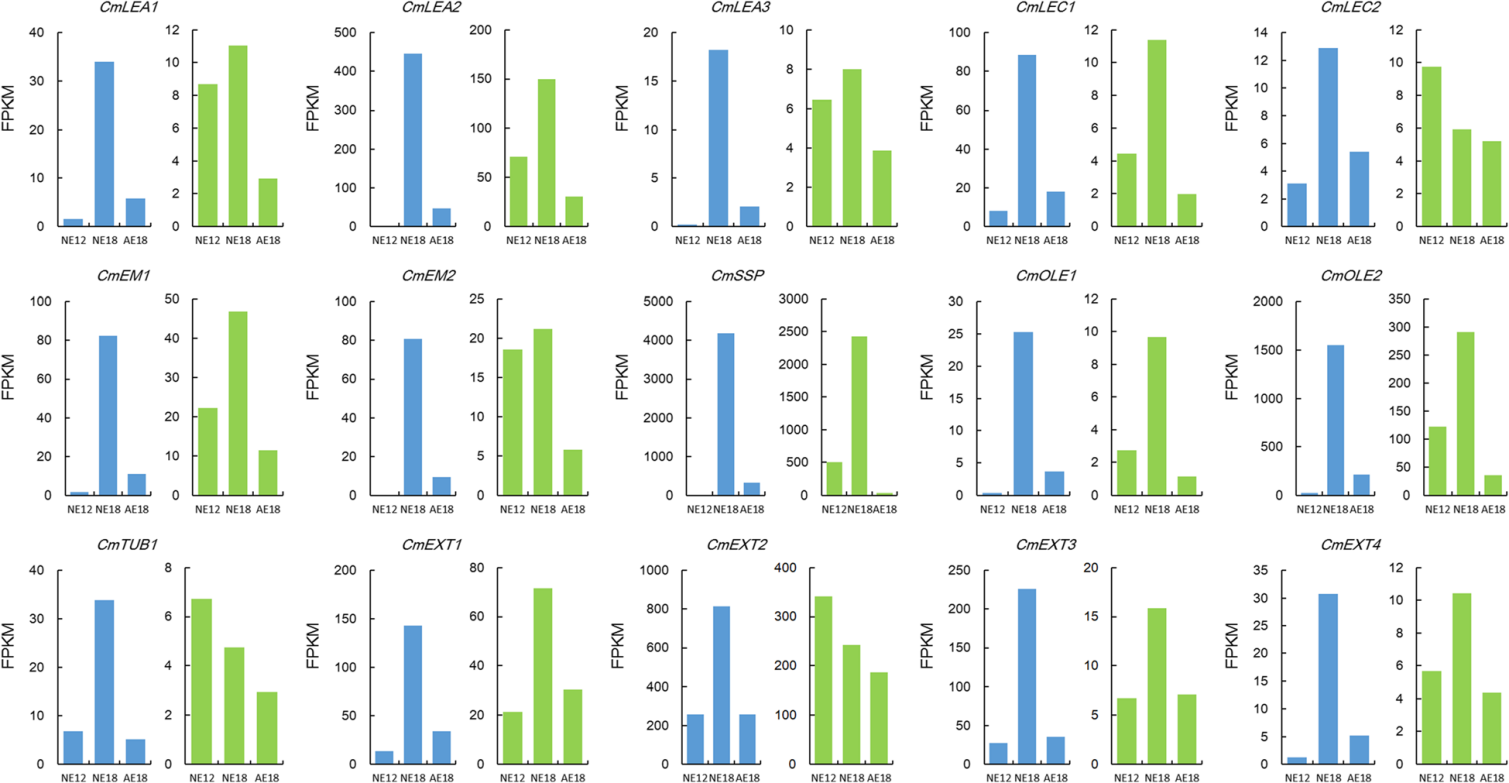
Analysis of the tricarboxylic acid (TCA) cycle pathway in normal and abnormal embryos 18 DAP in the cross *C. morifolium* × tetraploid *C. nankingense*. The map displays selected steps from the KEGG pathway ko00020 ‘Citrate cycle (TCA cycle)’. Yellow indicates higher relative levels and blue indicates lower levels in AE18. Enzymes are given as EC numbers: 1.2.4.1, pyruvate dehydrogenase; 1.8.1.4, dihydrolipoamide dehydrogenase; 2.3.1.12, dihydrolipoamide acetyltransferase; 2.3.3.1, citrate synthase; 1.3.5.1, succinate dehydrogenase; and 1.1.1.37, malate dehydrogenase

**Fig. 5:**
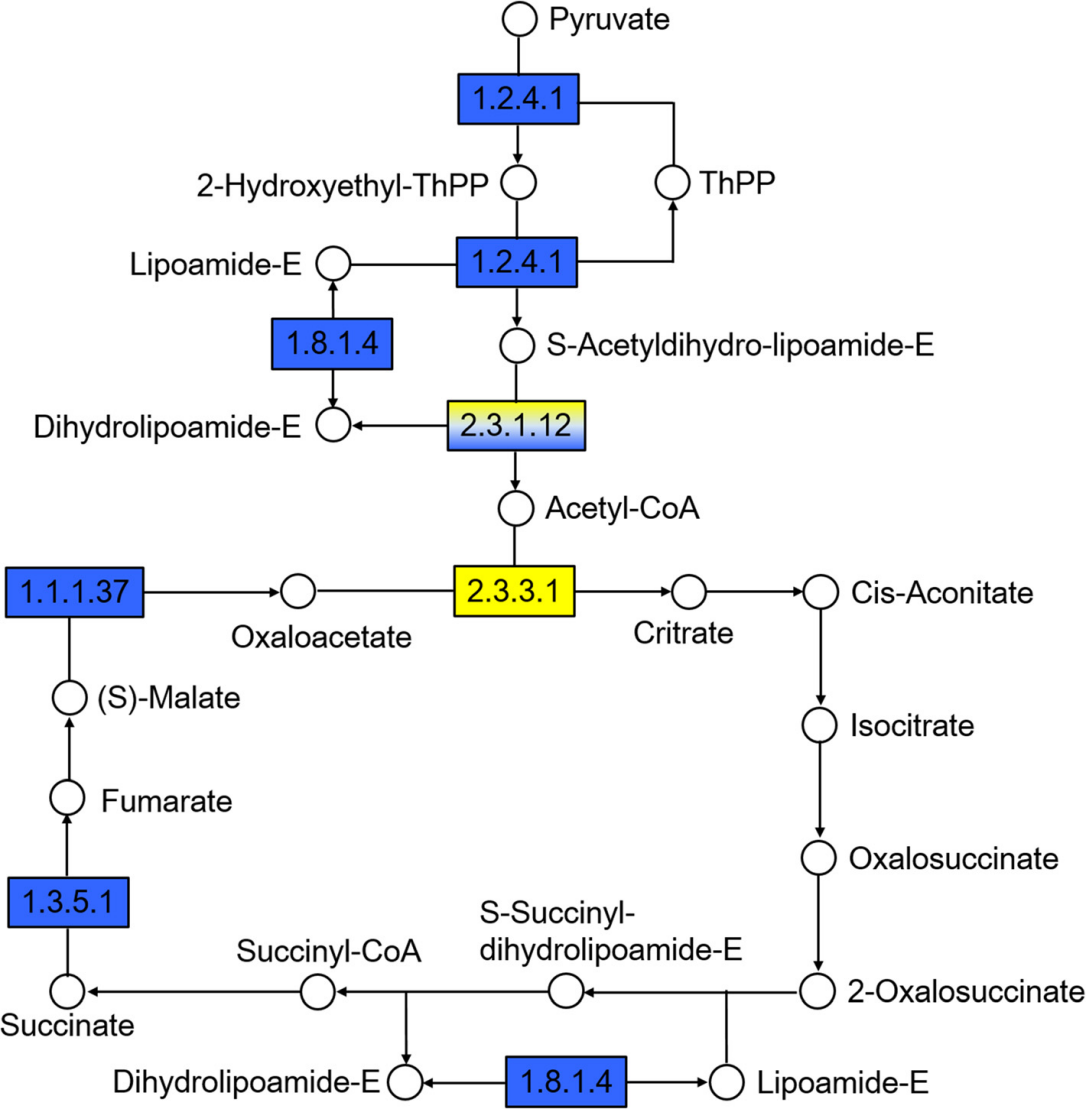
Analysis of the pathway related to auxin signal transduction in normal and abnormal embryos 18 DAP in the cross *C. morifolium* × tetraploid *C. nankingense*. The map displays selected steps from the KEGG pathway ko04075 ‘Plant hormone signal transduction’. Blue indicates the lower expression level of genes in AE18

KEGG is a database that analyzes gene products during metabolism and related gene functions involved in cellular processes. Using the KEGG database, we obtained two pathway annotations [energy metabolism (Fig. 4) and auxin signal transduction (Fig. 5)] between NE18 and AE18 in the cross *C. morifolium* × tetraploid *C. nankingense*. Compared with NE18, many of the enzymes involved in the pyruvate, acetyl-CoA and tricarboxylic acid (TCA) cycle pathways in the mitochondria were down-regulated in AE18; only citrate synthase [EC: 2.3.3.1] showed altered expression (Fig. 4). Auxin response factor (ARF) is the key factor in the auxin signal transduction pathway and receives auxin signals, leading to activation or repression of downstream genes. Two genes (*AUX*/*IAA* and *GH3*) involved in cell enlargement and plant growth reported to be regulated by ARF were down-regulated in the abnormal embryos when compared with the normal embryos (Fig. 5).

### qRT-PCR validation

**Fig. 6:**
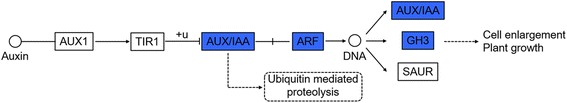
Validation of the RNA-Seq results by qRT-PCR. FPKM represents the gene abundance in the sequencing data of the transcriptome libraries

To validate the quality of the RNA-Seq data in this study, quantitative real-time RT-PCR (qRT-PCR) was performed on 29 randomly selected genes with differential expression levels. The differential expression patterns in the three samples, and the expression trend of almost all of the genes were consistent with the sequencing data (Fig. 6). Most of these genes were related to plant embryonic development (i.e., globulin seed storage protein, ethylene-responsive transcription factor, auxin-induced protein, embryonic protein, leafy cotyledon 1-like protein and senescence-related protein). Moreover, 40 DEGs (in Tables 5 and 6) in two crosses were validated, and 35 of 40 DEGs (87.5 %) showed the same type of altered expression as RNA-Seq (Additional file 5: Figure S3 and Additional file 6: Figure S4).

**Table 5:** Genes with same CDS sequences involved in embryo development at 12 DAP in two crosses

Gene ID (numbered in cross I)	FPKM in cross I	FPKM in cross II	UP/DOWN	Annotation	Acronym	Function
CL1478.Contig3	61.3065	6.9942	UP	Agamous-like MADS-box protein AGL80	*AGL80*	Affect female gametophyte and endosperm development in Arabidopsis [23]
Unigene25835	10.9284	0.509	UP	Floral homeotic protein APETALA2	*AP2*	Influence development of the zygotic embryo and endosperm [50]
CL4736.Contig1	15.3212	40.7673	DOWN	AP2 domain class transcription factor	*AP2*	Same as above
CL5669.Contig8	2.2975	4.874	DOWN	Receptor-like protein kinase HAIKU2	*Iku2*	Regulate the early endosperm cellularization and seed size [51]
Unigene26041	74.7454	28.9534	UP	Leucine-rich repeat extensin-like protein	*EXS*	Promote seed development and enhance cee size in *Arabidopsis* [52]
Unigene30982	39.5864	82.4367	DOWN	Multicopy suppressor of Ira1	*MSI1*	Be required for seed development [53]
CL15981.Contig1	18.8318	3.0993	UP	Auxin-binding protein 1	*ABP1*	Be required for cell elongation and division in Arabidopsis embryogenesis [54]
Unigene26808	54.5756	2.5485	UP	Arabidopsis histidine kinase	*AHK*	Serve as cytokinin receptors and regulate the seed size [55]
CL1615.Contig2	8.5299	3.0442	UP	Cytosolic phosphoglycerate kinase 2	*CPK2*	Be involved in glycolysis pathway [19]
Unigene744	24.4165	6.4023	UP	Citrate synthase	N/A	Be involved in the tricarboxylic acid (TCA) cycle pathway [19]
CL6259.Contig2	119.376	34.959	UP	Aconitase protein	N/A	Same as above
Unigene41988	8.1445	1.4722	UP	NADP-isocitrate Dehydrogenase	N/A	Same as above
CL5293.Contig2	22.4495	3.113	UP	2-succinylbenzoate-CoA ligase	N/A	Same as above
CL16042.Contig2	4.6659	1.2739	UP	Succinate dehydrogenase [ubiquinone] flavoprotein subunit 1	N/A	Same as above
CL12323.Contig3	46.8787	6.9274	UP	Malate dehydrogenase	N/A	Same as above
Unigene31171	1.0883	70.9733	DOWN	Late embryogenesis abundant protein	*LEA*	Be associated with desiccation tolerance during embryo maturation [29]
Unigene14917	6.9638	21.5178	DOWN	Senescence-related protein	N/A	Play essential roles of cell death during plant embryogenesis [56]
Unigene9288	48.7268	98.1867	DOWN	Programmed cell death protein	N/A	Same as above

Cross I: *C. morifolium* × tetraploid *C. nankingense*; Cross II: *C. morifolium* × diploid *C. nankingense*

*N/A* not applicable

**Table 6:** Genes with same CDS sequences involved in embryo development at 18 DAP in two crosses

Gene ID (numbered in cross I)	FPKM in cross I	FPKM in cross II	UP/DOWN	Annotation	Acronym	Function
Unigene33530	3.9624	1.4042	UP	Agamous-like MADS-box protein AGL80	*AGL80*	Affect female gametophyte and endosperm development [23]
Unigene42751	6.6841	13.8857	DOWN	Transcription factor APETALA2	*AP2*	Influence development of the zygotic embryo and endosperm [50]
CL9156	118.0731	314.5676	DOWN	AP2 domain class transcription factor	*AP2*	Same as above
CL13570.Contig2	7.4684	24.1844	Down	Receptor-like protein kinase HAIKU2	*Iku2*	Regulate the early endosperm cellularization and seed size [51]
CL11805.Contig1	10.291	1.3491	UP	Leucine-rich repeat receptor protein kinase EXS	*EXS*	Promote seed development in *Arabidopsis* [52]
CL1584.Contig1	1.7702	4.0557	DOWN	Multicopy suppressor of Ira1	*MSI1*	Be required for seed development [53]
Unigene7908	97.3165	4.6421	UP	Auxin-binding protein 1	*ABP1*	Be required for organized cell elongation and division in Arabidopsis embryogenesis [54]
Unigene7916	45.2634	11.3354	UP	Arabidopsis histidine kinase	*AHK*	Serve as cytokinin receptors and regulate the seed size [55]
CL4474	88.4354	5.6919	UP	Leafy cotyledon 1-like protein	*LEC1*	Regulate the embryogenesis, morphogenesis and seed maturation [25]
CL8636.Contig2	37.1208	7.9805	UP	Chloroplast phosphoglycerate kinase 3	*CPK*	Be involved in glycolysis pathway [19]
CL4864.Contig4	26.4753	2.0837	UP	ATP-citrate synthase beta chain protein 1-like	N/A	Be involved in the tricarboxylic acid (TCA) cycle [19]
CL13319	104.2401	8.611	UP	Citrate synthase	N/A	Same as above
CL2895.Contig3	66.6572	6.0815	UP	aconitase	N/A	Same as above
CL9180	112.6106	25.6244	UP	NADP-isocitrate dehydrogenase	N/A	Same as above
CL954.Contig1	33.9533	1.0652	UP	Succinyl-CoA ligase	N/A	Same as above
Unigene24208	110.71	12.2391	UP	succinate dehydrogenase [ubiquinone] iron-sulfur subunit 2	N/A	Same as above
Unigene30868	31.2681	5.6957	UP	Fumarate hydratase 1	N/A	Same as above
CL2079.Contig6	182.8558	25.9528	UP	Malate dehydrogenase	N/A	Same as above
Unigene31171	445.1903	150.025	UP	Late embryogenesis abundant protein	*LEA*	Be associated with desiccation tolerance during embryo maturation [29]
Unigene15940	0.9442	2.2972	DOWN	Senescence-induced receptor	N/A	Play essential roles of cell death during plant embryogenesis [56]
CL3148.Contig1	17.8887	73.7542	DOWN	Regulator of cell death	N/A	Same as above
Unigene9484	47.3966	12.4093	UP	Defender against cell death	N/A	Same as above

Cross I: *C. morifolium* × tetraploid *C. nankingense*; Cross II: *C. morifolium* × diploid *C. nankingense*

*N/A* not applicable

### Comparative analysis of identical genes during the same developmental stage between the two crosses

Based on the CDS sequence comparison between the two NE12 libraries from the two crosses, we identified 18 candidate genes (Table 5) that had not been analyzed in the *C. morifolium* × diploid *C. nankingense* cross, that might enhance chrysanthemum embryo development, including transcription factors, energy metabolism-related genes and other genes that might function in embryo development. Then, the same comparative analysis was applied to the NE18 libraries. A total of 22 candidates (Table 6) were found that facilitate the transformation from heart embryos to torpedo and cotyledonary embryos during seed development. These candidates were similar to NE12 except for unigene30868 (fumarate hydratase 1) and unigene9484 (defender against cell death), which were differentially expressed only at this stage. During both stages, we found that the auxin-binding protein, citrate synthase and other genes associated with energy metabolism were more highly expressed in cross I (*C. morifolium* × tetraploid *C. nankingense*) than in cross II (*C. morifolium* × diploid *C. nankingense*). Conversely, genes related to senescence and programmed cell death were down-regulated (Tables 5 and 6).

### Differentially-expressed proteins during chrysanthemum embryo development

In the embryos from cross I (*C. morifolium* × tetraploid *C. nankingense*), a total of 23 differentially expressed proteins (DEPs) (Table 7) were identified, 10 of which were considered predicted, hypothetical or unknown proteins, which was not useful for the elucidation of their functions. Thus, we aligned their peptides to the CDS sequences from the chrysanthemum embryo transcriptome library, and as a result, only two of the proteins remained unannotated, suggesting that studying the transcriptome was conducive to proteomics research for crops without genome sequences. Most of these DEPs were involved in energy metabolism (i.e., acetoacetyl CoA thiolase, pyruvate kinase, isopropylmalate synthase and malate dehydrogenase) and had high expression levels in normal embryos at 18 DAP.

**Table 7:** Differentially expressed proteins in normal and abnormal embryos in cross *C. morifolium* × tetraploid *C. nankingense*

Gene ID in transcriptome	Annotation in transcriptome	Annotation in proteome	Protein abundance			Accession	Peptides (95 %)	Species
			NE18:NE12	AE18:NE12	NE18:AE18			
CL11142.Contig2	acetoacetyl CoA thiolase	acetoacetyl CoA thiolase	1.54	1.22	1.25	gi|34597334	4	Helianthus annuus
CL12806.Contig2	GDP-mannose 3,5-epimerase 1 isoform 1	GDP-mannose 3’,5’-epimerase	0.76	0.54	1.42	gi|240248436	3	Solanum pennellii
CL12806.Contig2	GDP-mannose 3,5-epimerase 1 isoform 1	predicted protein	0.63	0.66	0.95	gi|224130650	3	Populus trichocarpa
CL13159.Contig2	ruBisCO large subunit-binding protein subunit alpha	hypothetical protein	0.77	0.51	1.51	gi|242032147	4	Sorghum bicolor
CL15574.Contig2	pyruvate kinase, cytosolic isozyme-like	Pyruvate kinase	1.57	0.69	2.26	gi|92870921	3	Medicago truncatula
CL2359.Contig5	putative isopropylmalate synthase	putative isopropylmalate synthase	1.26	1.52	0.82	gi|193290704	2	Capsicum annuum
CL4538.Contig4	glyceraldehyde 3-phosphate dehydrogenase	glyceraldehyde-3-phosphate dehydrogenase	1.48	1.64	0.90	gi|83701234	5	Talipariti tiliaceum
CL4750.Contig2	predicted protein	hypothetical protein	1.86	0.91	2.04	gi|242074456	2	Sorghum bicolor
CL4761.Contig1	photosystem II cp47 protein	photosystem II 47 kDa protein	1.53	1.38	1.11	gi|75755685	4	Acorus calamus
CL480.Contig6	Transitional endoplasmic reticulum ATPase	predicted protein	1.12	0.43	2.62	gi|326527541	6	Hordeum vulgare subsp. vulgare
CL4814.Contig3	unknown	unnamed protein product	0.65	0.87	0.75	gi|257674447	4	Helianthus annuus
CL5465.Contig4	elongation factor 1-gamma-like	hypothetical protein	1.51	1.56	0.97	gi|225465198	4	Vitis vinifera
CL7034.Contig6	putative UDP-glucose dehydrogenase 1	hypothetical protein	0.81	1.54	0.53	gi|302779800	3	Selaginella moellendorffii
CL8744.Contig1	14-3-3-like protein	14-3-3 protein 6	1.19	1.62	0.73	gi|26454608	7	SOLLC
Unigene11968	thiazole biosynthetic enzyme	unknown	0.86	0.63	1.36	gi|116784521	3	Picea sitchensis
Unigene12985	thioredoxin peroxidase	peroxiredoxin	1.53	1.04	1.47	gi|300078580	2	Jatropha curcas
Unigene15015	Nucleoside diphosphate kinase	cytosolic nucleoside diphosphate kinase	1.52	0.81	1.87	gi|73808794	4	Solanum chacoense
Unigene16290	calcium-dependent protein kinase 1	unnamed protein product	0.58	0.57	1.02	gi|257734686	2	Solanum lycopersicum
Unigene23495	serine carboxypeptidase precursor	putative serine carboxypeptidase precursor	1.74	1.13	1.54	gi|18447763	5	Gossypium hirsutum
Unigene30534	sucrose sucrose 1-fructosyltransferase	sucrose:sucrose 1-fructosyltransferase	0.59	0.64	0.92	gi|162424641	5	Lactuca sativa
Unigene45009	tubulin beta chain, putative	tubulin beta-9 chain	0.81	0.61	1.32	gi|7268885	12	Arabidopsis thaliana
Unigene7304	malate dehydrogenase, mitochondrial-like	malate dehydrogenase	1.64	1.52	1.08	gi|7798706	5	Vitis vinifera
Unigene8567	conserved hypothetical protein	predicted protein	1.90	2.83	0.67	gi|224138258	2	Populus trichocarpa

To analyze the expression levels of these 23 DEPs in cross II (*C. morifolium* × diploid *C. nankingense*), peptides were searched in a proteomic library*.* As a result, 10 proteins were aligned with the same peptides, and their expression levels were variable. The proteins associated with energy metabolism, such as pyruvate kinase (gi|92870921), transitional endoplasmic reticulum ATPase (gi|326527541) and cytosolic nucleoside diphosphate kinase (gi|73808794), had significantly higher NE18/AE18 ratios in cross I compared with those of cross II (Table 8).

**Table 8:** Differentially expressed proteins with same peptides during chrysanthemum embryo development in two crosses

Protein name	Protein abundance in Cross I			Protein abundance in Cross II			Accession	Species
	NE18:NE12	AE18:NE12	NE18:AE18	NE18:NE12	AE18:NE12	NE18:AE18		
acetoacetyl CoA thiolase	1.54	1.22	1.25	no hit			gi|34597334	Helianthus annuus
GDP-mannose 3’,5’-epimerase	0.76	0.54	1.42	no hit			gi|240248436	Solanum pennellii
predicted protein	0.63	0.66	0.95	no hit			gi|224130650	Populus trichocarpa
hypothetical protein	0.77	0.51	1.51	no hit			gi|242032147	Sorghum bicolor
pyruvate kinase	1.57	0.69	2.26	1.04	0.74	1.41	gi|92870921	Medicago truncatula
putative isopropylmalate synthase	1.26	1.52	0.82	no hit			gi|193290704	Capsicum annuum
glyceraldehyde-3-phosphate dehydrogenase	1.48	1.64	0.9	1.05	1.29	0.81	gi|83701234	Talipariti tiliaceum
hypothetical protein	1.86	0.91	2.04	no hit			gi|242074456	Sorghum bicolor
photosystem II 47 kDa protein	1.53	1.38	1.11	no hit			gi|75755685	Acorus calamus
predicted protein	1.12	0.43	2.62	1.12	1.07	1.05	gi|326527541	Hordeum vulgare subsp. vulgare
unnamed protein product	0.65	0.87	0.75	0.68	1.11	0.61	gi|257674447	Helianthus annuus
hypothetical protein	1.51	1.56	0.97	no hit			gi|225465198	Vitis vinifera
hypothetical protein	0.81	1.54	0.53	1.03	0.89	1.16	gi|302779800	Selaginella moellendorffii
14-3-3 protein 6	1.19	1.62	0.73	0.91	1.06	0.86	gi|26454608	SOLLC
unknown	0.86	0.63	1.36	no hit			gi|116784521	Picea sitchensis
peroxiredoxin	1.53	1.04	1.47	no hit			gi|300078580	Jatropha curcas
cytosolic nucleoside diphosphate kinase	1.52	0.81	1.87	0.77	1.12	0.68	gi|73808794	Solanum chacoense
unnamed protein product	0.58	0.57	1.02	no hit			gi|257734686	Solanum lycopersicum
putative serine carboxypeptidase precursor	1.74	1.13	1.54	no hit			gi|18447763	Gossypium hirsutum
sucrose:sucrose 1-fructosyltransferase	0.59	0.64	0.92	1.08	0.99	1.09	gi|162424641	Lactuca sativa
tubulin beta-9 chain	0.81	0.61	1.32	0.77	0.56	1.37	gi|7268885	Arabidopsis thaliana
malate dehydrogenase	1.64	1.52	1.08	1.02	0.99	1.03	gi|7798706	Vitis vinifera
predicted protein	1.9	2.83	0.67	no hit			gi|224138258	Populus trichocarpa

Cross I: *C. morifolium* × tetraploid *C. nankingense*; Cross II: *C. morifolium* × diploid *C. nankingense*

no hit: These proteins in cross II have not been retrieved in cross I by their peptide.

## Discussion

Polyploid breeding can improve breeding efficiency by adjusting the optimal chromosome number to successfully match in interspecific crosses [17]. In this study, a few seeds were obtained following hybridization between a hexaploid maternal parent and a tetraploid male parent. Conversely, the cross with the diploid male failed to produce seeds [16], suggesting that the ploidy of the male parent might affect the formation of hybrid plants. The closer the chromosome ploidy of the parents, the more likely the cross will be successful. The same phenomenon has been previously reported in chrysanthemum interspecific hybridization, in which the cross *C. morifolium* × *C. nankingense* failed, and cross ability was greatly affected by the pollen grains on stigmas and embryo abortion [10]. In another cross, *C. morifolium* × diploid *C. nankingense*, six hybrids were created, but they required ovary rescue, suggesting that the post-fertilization barrier played an important role in embryo abortion [8]. In the previous study in which *C. morifolium* ‘Zhongshanzixing’ was the female parent, the cross using diploid *C. nankingense* failed to produce seeds, whereas hybrids were obtained when the male parent was a tetraploid *C. nankingense* [9]. Therefore, the doubled chromosome number of *C. nankingense* likely enabled a successful interspecific cross in the cultivated chrysanthemum.

Currently, high-throughput sequencing technology has been used to study embryo abortion research [14, 16] as well as for de novo genome assembly, molecular marker and genome diversity studies, the discovery of novel genes and investigations into gene expression patterns. Therefore, studying the genes or proteins related to embryo or endosperm development in various crosses is also helpful.

### Transcriptome and proteome data analysis during chrysanthemum embryo development

In this study, COG and GO functional annotations were performed. In the COG functional annotation (Additional file 1: Figure S1) from the three transcriptomic libraries, the top five of the 25 obtained COG categories were same as cross II [16]. Interestingly, the same situation was observed in the GO functional annotation (Additional file 2: Figure S2), suggesting that the genes involved in the regulation of these processes had widespread expression during chrysanthemum embryonic development. Next, we detected 3276 DEGs (Fig. 2) and 23 DEPs (Table 7) between the normal and abnormal embryos at 18 DAP that might be related to embryo and endosperm development in this cross. Finally, using KEGG annotation, two pathways were chosen in NE18 and AE18: ‘Citrate cycle (TCA cycle)’ and ‘Plant hormone signal transduction’. Between two crosses, qRT-PCR confirmed the reliability of the 35 DEGs in Tables 5 and 6. Study has shown that the DEGs detection depended on the pooled samples or individual samples [18]. Thus, pooled samples were used in this study, which might have caused the false positivity observed for the other 5 DEGs. RNA-Seq of the individual samples is a better way to detect DEGs. However, because of the difficulty collecting material and the limitation of florescence, pooled samples are also worthwhile when qRT-PCR verification is used as a complementary approach to exclude false positive of DEGs.

### Expression of genes involved in energy metabolism pathways

Energy metabolism is necessary for nearly all biological process, including plant embryo development. In our results, the KEGG pathway analysis between NE18 and AE18 in cross I demonstrated the importance of energy metabolism for normal embryo development. Most of the enzymes involved in the TCA cycle exhibited reduced expression levels in abnormal embryos at 18 DAP (Fig. 4), suggesting that decreased energy was not sufficient for continued embryo growth and resulted in abortion. The genes and proteins involved in energy metabolism (especially in cytosolic glycolysis and the mitochondrial TCA cycle) were identified as differentially expressed during embryogenesis (Fig. 4, Tables 5, 6, 7, 8). In normal embryos at 12 DAP in the two crosses, the genes related to citrate synthase, aconitase protein, NADP-isocitrate dehydrogenase and malate dehydrogenase were up-regulated in cross I (Table 5); moreover, normal embryos at 18 DAP exhibited the same expression pattern, whereas the genes associated with the TCA cycle and glycolysis pathway in cross I were also more highly expressed (Table 6). During maize embryo development, genes related to metabolism showed altered expression; these gene transcripts accumulated at higher levels between 10 and 20 DAP during the active process of metabolism [19]. Using comparative proteome analysis, we identified some proteins associated with energy metabolism, such as pyruvate kinase (the ratios of NE18/AE18 for cross I and II were 2.26 and 1.41, respectively) and the transitional endoplasmic reticulum ATPase (the ratios were 2.62 and 1.65, respectively). In this study, the higher expression of these genes in NE12 and NE18 and the obviously higher ratios (NE18/AE18) of these proteins in cross I (Table 8) suggested that more active energy metabolism occurred in cross I during the development of chrysanthemum embryos, which would help provide sufficient energy for improved embryo development and reduced rates of abortion, which increased the production of seeds.

### Expression of genes involved in hormonal signaling

Plant hormones, such as auxins, cytokinins, and gibberellins are involved in the regulation of seed development [20]. For example, in an auxin-binding protein 1 (*abp1*) mutant, the embryos develop abnormally after the globular embryo stage because of a failure of cell elongation [21]. In NE12 and NE18 of the two crosses, the expression of *ABP1* was significantly higher in cross I (Tables 5 and 6), suggesting that *ABP1* might facilitate normal embryo development in chrysanthemums by mediating auxin-induced cell elongation and cell division. In cross I, the KEGG pathway related to auxin signal transduction indicated down-regulated expression of the DNA binding ARF activators (Fig. 5), which were shown to regulate the expression of auxin-responsive genes. These auxin-responsive genes belong to three major groups: *Aux/IAA*, *Small Auxin Up RNA* (*SAUR*) and *Gretchen Hagen 3* (*GH3*) [22]. In abnormal embryos, the expression of *Aux/IAA* and *GH3* were down-regulated, indicating that these genes play an important role during normal embryo development in chrysanthemum.

### Expression of transcription factors

Transcription factors (TFs) are critically important during plant flower and fruit development. Several types of TFs were differentially expressed during chrysanthemum embryo development in the two crosses. However, how these TFs are regulated from early embryogenesis to maturity is unknown. Transcriptome analysis revealed that some of the transcription factors were significantly up-regulated in cross I, such as *AGAMOUS-LIKE62* (*AGL62*), *AGL80*, *exs* and *LEAFY COTYLEDON1* (*LEC1*), which enhance important functions necessary for in plant embryonic development. In *Arabidopsis*, the *AGL80* has been demonstrated to be involved in endosperm development. In *fem111* plants, female gametophytes contain a T-DNA insertion in *AGL80*, resulting in the reduced size of the central cell’s nucleolus and vacuole and a failure to generate endosperm [23]. In NE12, the expression level of *AGL80* (CL1478.Contig3) in cross I was far greater than in cross II (Table 5). In *Arabidopsis*, *AGL80* was expressed exclusively in the endosperm at the stage from 8 to 72 h after pollination, and the expression level was strongest in young seeds, decreasing gradually as the seeds age [23]. Similar gene expression was observed in this study, in which the expression of *AGL80* (Unigene33530) decreased in NE18 compared to NE12 (Table 6). The globular-to-heart transition stage is essential for endosperm development and provides nutrient supplies for embryo development [20]. Thus, the high expression of *AGL80* at 12 DAP observed in our study might promote normal chrysanthemum endosperm development by ensuring the energy supply for embryo development at 18 DAP, suggesting that *AGL80* in cross I was required for endosperm development and the improved success rate of the interspecific cross.

Another indispensable transcription factor involved in embryogenesis is *LEC1. LEC* regulates embryo development and is required for normal development during the morphogenesis and maturation stages in *Arabidopsis* [24, 25]. Studies on the functions of *LEC* demonstrated that *LEC* was required to specify the suspensor cell fate and cotyledon identity during early embryogenesis [26, 27]. However, during late embryogenesis, *LEC* was required for the acquisition of desiccation tolerance and the expression of many maturation-specific genes [28]. Our findings showed that *LEC1* was expressed mainly in cross I, especially in NE18 (Fig. 3 and Table 6), and was significantly reduced in cross II (Table 6). However, there was no significant difference in NE12 between the two crosses. This *LEC1* expression suggested it might be a transcriptional regulator of chrysanthemum seed development and required for normal early stage embryo development. Moreover, a deficiency in *LEC1* during the late development stage might cause the seeds to lose their desiccation tolerance, resulting in abnormal development or abortion in the *C. morifolium* × diploid *C. nankingense* cross.

### Expression of other genes related to embryo development

Plant embryogenesis is a complicated process regulated by numerous genes and regulators. Moreover, various proteins are synthesized steadily, such as late embryogenesis abundant (LEA) protein, embryonic protein, oleosin and proteins related to senescence or cell death. In plants, the LEA protein is associated with desiccation tolerance during embryo maturation when seeds undergo a developmentally regulated dehydration period. A study found that an *A. thaliana* mutant with a T-DNA insertion allele of the *AtEM6* gene belonging to the group 1 *LEA* family might be required for normal seed development. Studies on regulation and expression patterns indicated that the *LEA* genes in diverse species, such as rice, barley and soybean, were primarily expressed in developing or mature embryos [29], whereas *OsLEA1a* transcripts accumulated to high levels in dried mature embryos [30]. Here, *LEA* expression in NE18 from cross I was higher than that from cross II (Fig. 3), suggesting that the LEA proteins were required for normal seed development and expressed primarily at the late embryonic stage, when they might function as a buffer to protect from the water loss that occurs during embryo maturation. Therefore, more LEA proteins were expressed at NE18 when tetraploid *C. nankingense* was the male parent, which might be one reason that we were able to obtain seeds.

In dicotyledons, seed storage proteins (SSPs) mainly accumulate in the endosperm [31], including the 7S and 11S globulin classes [32]. Arabidopsis *lec* mutants exhibit defective synthesis and the accumulation of specific storage molecules, including SSPs [28]. Additionally, oleosins, which are seed-specific lipid storage proteins, have specific functions in seed tissues controlling oil body structure and lipid accumulation [33]. We showed that the expression levels of the embryonic protein (*CmEM*), 11S globulin seed storage protein (*CmSSP*) and oleosin (*CmOLE*) were all most abundant in NE18 in cross I, and their expression in both NE12 and NE18 was substantially higher than that in cross II (Fig. 3). This analysis provides evidence that more storage proteins accumulated quickly during the phase from 12 – 18 DAP when the interspecific cross occurred between the hexaploid *C. morifolium* and tetraploid *C. nankingense*. Thus, these specific proteins might contribute to the completion of embryonic development and maturation.

In contrast, particular genes and proteins related to senescence or cell death had different expression patterns and were down-regulated in cross I. Previous evidence indicated that programmed cell death occurred concomitantly with the development of the embryo and endosperm [16, 34, 35]. In this study, genes related to programmed cell death in NE12 (Table 5) and the senescence-induced receptor and regulator of cell death in NE18 (Table 6) were down-regulated in the *C. morifolium* × tetraploid *C. nankingense* cross. However, the gene defender against cell death (Unigene9484) was up-regulated, suggesting that chromosome doubling of the male parent might epigenetically inhibit the expression of senescence-induced genes and cause normal embryo development.

## Conclusions

We sequenced and characterized the transcriptome and proteome of normal and abnormal chrysanthemum ovules and analyzed the differentially expressed genes and proteins associated with embryogenesis in two interspecific crosses in which the female was diploid or tetraploid. The comparative analysis of the two crosses demonstrated important roles for energy metabolism, auxin signal transduction, transcription factors and proteins related to cell death during chrysanthemum embryo development. The results provide valuable evidence at the molecular level that doubling the chromosome number in *C. nankingense* might overcome the distant hybridization barrier in the cultivated chrysanthemum.

## Methods

### Plant materials and artificial hybridization

**Fig. 7:**
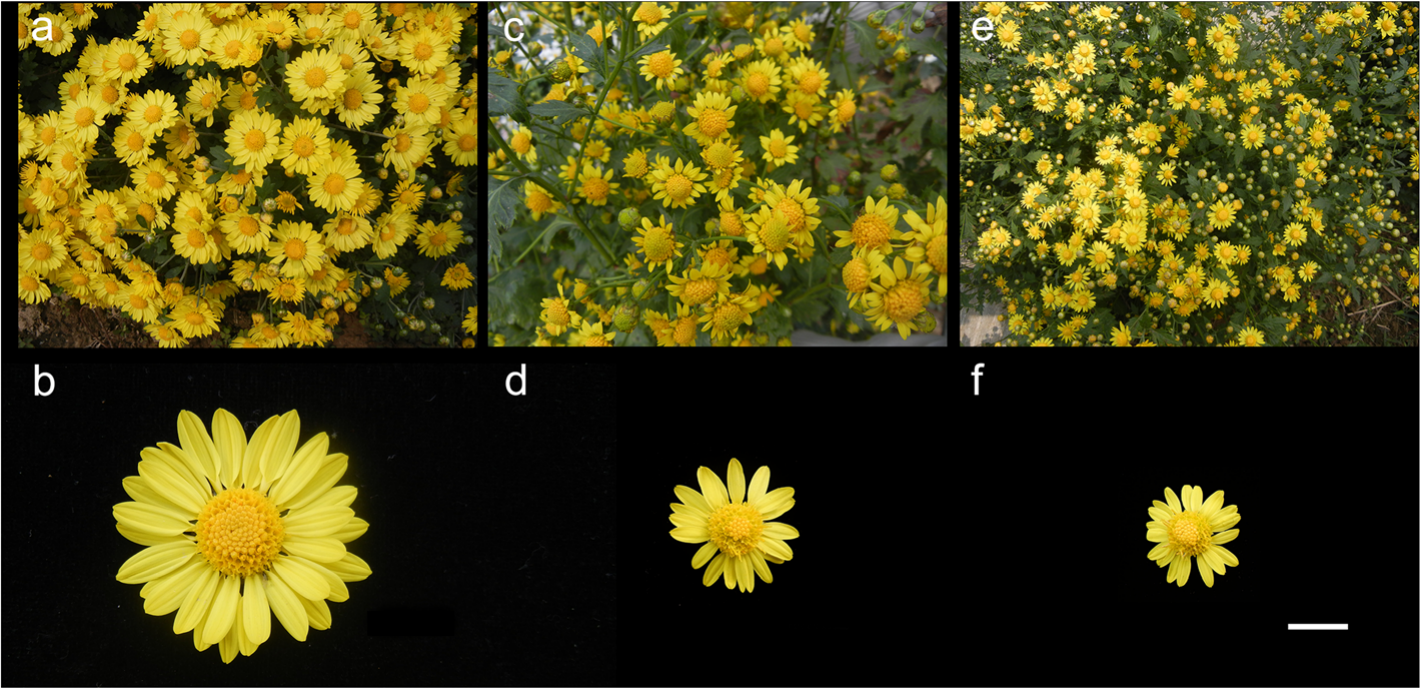
Flower morphology of *C. morifolium* ‘Yuhualuoying’ tetraploid *C. nankingense* and diploid *C. nankingense*. **a**, **b** *C. morifolium* ‘Yuhualuoying’. **c**, **d** Tetraploid *C. nankingense*. **e**, **f** Diploid *C. nankingense*. Bar = 1 cm

The chrysanthemum ‘Yuhualuoying’ (2n = 6X = 54) (Fig. 7a, b) is a ground-cover cultivar with desirable ornamental traits for landscape applications. The tetraploid *C. nankingense* (2n = 4X = 36) (Fig. 7c, d) has a stronger tolerance to both abiotic and biotic stresses and is an autopolyploid generated by colchicine doubling of the diploid *C. nankingense* (2n = 2X = 18) [36] (Fig. 7e, f). The plants were grown in the Chrysanthemum Germplasm Resource Preserving Center, Nanjing Agricultural University, China. The interspecific cross *C. morifolium* × tetraploid *C. nankingense* was performed according to a previously reported method [37]. For abundant and high quality samples, we planted a total of 60 chrysanthemum plants in three areas to generate approximately 1500 inflorescences (18-23 female ligulate florets or ray florets containing one ovule per inflorescence) for artificial hybridization. At the same time, the interspecific cross *C. morifolium* × diploid *C. nankingense* was performed with approximately 100 inflorescences to generate the seed setting rate statistics. Statistical analyses for the seed setting rate was performed with a one-way analysis of variance using SPSS version 20.0 (IBM SPSS Statistics, IBM Corporation, Chicago, IL), and the means were compared using Student’s *t*-test with alpha = 0.05.

### Sample preparation and RNA extraction

We collected chrysanthemum ovules during two developmental stages. At 12 DAP, almost all of the embryos were normal; therefore, we collected 0.6 g of normal ovules from each of the three planted areas, (0.2 g was stored separately, and the remaining three 0.4 g samples were mixed for NE12). Thereafter, the proportion of normal embryos was gradually reduced concomitant with embryonic development. At 18 DAP, some embryos reached the heart-shaped embryo stage, whereas others underwent degeneration; therefore, we collected more than 1.0 g of normal and abnormal ovules identically to NE12. All of the samples were immediately frozen in liquid nitrogen and stored at -80 °C.

Meanwhile, for TEM observation, we collected the ovules from NE12, NE18 and AE18 from cross I, and immediately immersed them in 2.5 % (v/v) glutaraldehyde (in 0.1 mol/L phosphate buffer, pH 7.2), gently extracted the air using a syringe, and then stored the sample at 4 °C. Next, the ovules were washed five times in the same phosphate buffer and post-fixed in 1.5 % osmium tetroxide for 5 h. Then the ovules were treated in graded PHEM buffer (60 mmol/L pipes; 25 mmol/L Hepes; 10 mmol/L EGTA; 2 mmol/L MgCl2; pH 7. 0) and 100 % ethanol, and then embedded in Epon 812. The sections were cut into 80 nm thickness using an LKB-V ultramicrotome (Bromma, Sweden) and stained with uranyl acetate and lead citrate. Finally, the sections were imaged under a transmission electron microscope (Hitachi H-7650) at 80 kV [38]. Total RNA was isolated using TRIzol reagent according to the manufacturer’s protocol (Takara Bio Inc., Otsu, Japan). The RNA quantity and quality were determined using an Agilent 2100 RNA 6000 Kit (Agilent Technologies, Santa Clara, CA, USA) and electrophoresis on a 1 % agarose gel.

### cDNA preparation and Illumina deep sequencing

After RNA extraction and DNase I treatment, mRNA was isolated using magnetic beads with Oligo (dT) and fragmented in mixed fragmentation buffer. Then cDNA was synthesized using the mRNA fragments. Short fragments were purified and resolved with EB buffer for end repair and single nucleotide A (adenine) addition. Then, the short fragments were connected with adapters, and the suitable fragments were selected for PCR amplification. The Agilent 2100 Bioanalyses and ABI StepOnePlus Real-Time PCR System were used in quantification and qualification of the sample library [14]. At last, the libraries were sequenced using Illumina HiSeq™ 2000 according to the manufacturer’s instructions (Illumina, San Diego, CA, USA) [39] at the Beijing Genomics Institute [(BGI)-Shenzhen, Shenzhen, China; http://www.genomics.cn/index.php].

### Filtering raw reads and de novo assembly

The raw reads produced from sequencing contain adapters and unknown or low-quality bases. Therefore, obtaining clean reads by removing the unqualified reads is necessary. Then, transcriptome de novo assembly was performed with Trinity software [39]. The TGICL program (version 2.0; http://sourceforge.net/projects/tgicl) was used for de novo assembly of the data by Trinity. This program can splice and remove redundant unigenes from each sample’s assembly to acquire non-redundant unigenes that are as long as possible. Then, the unigenes were divided into two classes by gene family clustering. The first clusters were denoted by the prefix CL and the suffix indicating the cluster number containing several similar unigenes (more than 70 %) in one cluster. The other cluster contained singletons with the prefix representing the unigene.

In the final step, a BlastX alignment (E-value < 0.00001) was performed between databases such as NR, Swiss-Prot, KEGG and COG, and the directions of the unigenes were decided according to the best alignment results. If the results conflicted in the different libraries, the sequence direction was determined by the priority order of NR, Swiss-Prot, KEGG and COG. If a unigene was not aligned to any of the above databases, the software ESTScan [40] was used to predict the sequence direction.

### Unigene functional annotation

First, unigene sequences were aligned to protein databases including NR, Swiss-Prot, KEGG and COG by BlastX and to the nucleotide database NT by BlastN. Then, the putative functional annotations of the unigenes were retrieved. Next, we used the Blast2GO program [40] to obtain gene ontology (GO) (http://www.geneontology.org) annotations of the unigenes based on the NR annotations. After GO annotations were obtained for each unigene, WEGO software [41] was used to obtain GO functional classifications for all unigenes. GO has three ontologies: molecular function, cellular component and biological process. Finally, we used the KEGG database to study the genes’ functions in cellular processes and obtain pathway annotation for the unigenes based on KEGG annotation.

### Differential unigene expression analysis

To predict unigene expression levels in different samples, we calculated the unigene expression levels using FPKM [42]. After the calculation, rigorous algorithms were used to identify differentially expressed genes between the two samples by referring to the Audic and Claverie’s method [43]. In our analysis, these DEGs met the criteria of an FDR ≤ 0.001 and ratio larger than 2. Then, GO functional analysis and KEGG pathway analysis were performed for the DEGs.

In the GO functional analysis, all of the DEGs were mapped to each term of the GO database and the gene numbers associated with each GO term were calculated. After obtaining a gene list and gene numbers for each included GO term, we used the hypergeometric test to identify significantly enriched GO terms in the DEGs compared to the genome background. In KEGG pathway analysis, pathway enrichment analysis identifies significantly enriched metabolic pathways or signal transduction pathways that involve the DEGs compared with the whole genome background.

### Quantitative real-time PCR analysis

To ensure the libraries’ reliability, we randomly selected 29 differentially expressed genes and validated the data by qRT-PCR using three biological replicate samples. The qRT-PCR assays were conducted as described by Song et al [44] on a Mastercycler ep realplex device (Eppendorf, Hamburg, Germany). In addition, qRT-PCR also performed on the 40 DEGs shown in Tables 5 and 6 using three biological replicates from two crosses. Gene-specific primers (sequences shown in Additional file 7: Table S3) were designed using PRIMER3 RELEASE 2.3.4 [45]; the reference sequence for the quantitative expression analysis was the *Elongation Factor 1a* (*EF1a*) gene, which is stably expressed in chrysanthemum [16, 46]. Relative transcript abundances were calculated using the 2^−ΔΔCt^ method [47].

### Protein preparation, sample labeling and iTRAQ analysis

For each sample, approximately 0.5 g of ovules was used for protein extraction by the trichloroacetic acid/acetone method [48]. The extracted proteins were acetone precipitated and redissolved in dissolution buffer [16]. The protein concentration was determined according to the Bradford assay. Prior to digestion, 100 μg of protein from each sample was denatured and alkylated and the cysteines were blocked according to the 8-plex iTRAQ reagent kit instructions (Applied Biosystems, California, USA). Then, the protein was digested [16]. The NE12, NE18 and AE18 samples were labeled with 113, 119, and 121 iTRAQ tags, respectively. The three labeled samples from each individual replicate experiment were combined and vacuum-dried. The pooled sample was resuspended in the SepPac™ C18 cartridge (1 cm^3^/50 mg, Waters Corporation, Milford, MA, USA) to remove the salt buffer and re-dissolved in a low salt buffer [16]. Then, the proteins were separated by Poly-LC strong cation exchange chromatography using a PolySULFOETHYL A (2.1 × 100 mmi.d.) HPLC column (PolyLC Inc, Columbia, MD, USA). The peptide mixture was collected using a high salt buffer [16] for elution. After the acetonitrile was volatilized, excess KCl was removed by the SepPac™ C18 cartridge, and the sample was dried in a vacuum concentrator.

Next, the peptide mixture was re-dissolved in buffer A [16] and fractionated by high pH separation using a Shimazu UFLC system (Shimazu, Kyoto, Japan). The high-pH separation was performed using a four-step linear gradient (5 % B for 30 min, 35 % B for 30 min, 80 % B for 2 min, and 5 % B for 2 min; B, 20 mM NH_4_HCO_2_ in 90 % acetonitrile, pH 10.0, adjusted with NH_4_OH). The peptides were eluted from the LC column at a flow rate of 200 μL/min. A total of 15 fractions were collected and dried in a vacuum concentrator for the next step.

### Mass spectrometry analysis

Mass spectrometric analysis of the iTRAQ-labeled samples was performed on a Nano Aquity UPLC system (Waters Corporation, Milford, MA, USA) connected to an LTQ Orbitrap XL mass spectrometer (Thermo Electron Corp., Bremen, Germany) equipped with an online nanoelectrospray ion source (Michrom Bioresources, Auburn, CA, USA). The detailed steps were described previously [16].

### Database searches and analysis

Protein identification and quantification for the iTRAQ experiment were performed with ProteinPilot software version 4.0 (Applied Biosystems) [49]. The resulting dataset was auto-bias corrected to remove any variations caused by unequal mixing when combining differently labeled samples. For iTRAQ quantitation, the peptide for quantification was automatically selected by the Pro Group algorithm (at least two peptides with 99 % confidence) to calculate the reporter peak area, error factor (EF), and p-value. For the selection of differentially expressed proteins, we considered the following situations: (1) the proteins must contain at least two unique high-scoring peptides and (2) the proteins must have a *p*-value < 0.05, and the proteins identified with mass tag changes ratio must be ≥ 1.3 or ≤ 0.75.

## Abbreviations

AE18, abnormal embryo at 18 DAP; ARF, auxin response factor; COG, clusters of orthologous groups of proteins; DAP, days after pollination; DEGs, differentially expressed genes; DEPs, differentially expressed proteins; FPKM, fragments per kb per million fragments; GO, gene onotology; KEGG, Kyoto Encyclopedia of genes and genomes; NE12, normal embryo at 12 DAP; NE18, normal embryo at 18 DAP; qRT-PCR, quantitative real-time PCR; TCA, tricarboxylic acid cycle; TEM, transmission electron microscopy

## Additional files

Additional file 1: Figure S1. Clusters of orthologous group (COG) function classification of the embryo transcriptome. The 20,391 sequences were grouped into 25 categories. (TIF 1297 kb) Additional file 2: Figure S2. Gene Ontology (GO) classification of the embryo transcriptome. The transcriptome datasets were grouped into three GO classifications: biological process, cellular component and molecular function. (TIF 380 kb) Additional file 3: Table S1. The 128 KEGG pathways. (DOCX 22 kb) Additional file 4: Table S2. Differential expression genes in NE12, NE18 and AE18. (XLS 12079 kb) Additional file 5: Figure S3. qRT-PCR validation of the DEGs involved in embryo development at 12 DAP in two crosses. (TIF 165 kb) Additional file 6. Figure S4. qRT-PCR validation of the DEGs involved in embryo development at 18 DAP in two crosses. (TIF 204 kb) Additional file 7: Table S3. Primer sequences for qRT-PCR. (DOCX 17 kb)
