# Erratum to: Chromosome doubling to overcome the chrysanthemum cross barrier based on insight from transcriptomic and proteomic analyses

**DOI:** 10.1186/s12864-016-3071-x

**Published:** 2016-09-07

**Authors:** Fengjiao Zhang, Lichun Hua, Jiangsong Fei, Fan Wang, Yuan Liao, Weimin Fang, Fadi Chen, Nianjun Teng

**Affiliations:** 1 College of Horticulture, Nanjing Agricultural University, Nanjing, 210095 China; 2 Jiangsu Province Engineering Lab for Modern Facility Agriculture Technology and Equipment, Nanjing, 210095 China

## Erratum

Unfortunately, the original version of this article [1] contained an error. The Figure legends for Figs. 3, 4, 5 and 6 are incorrect. The correct version of Figs. 3, 4, 5 and 6 can be found below.

The correct match is as follows:

**Fig. 3:**
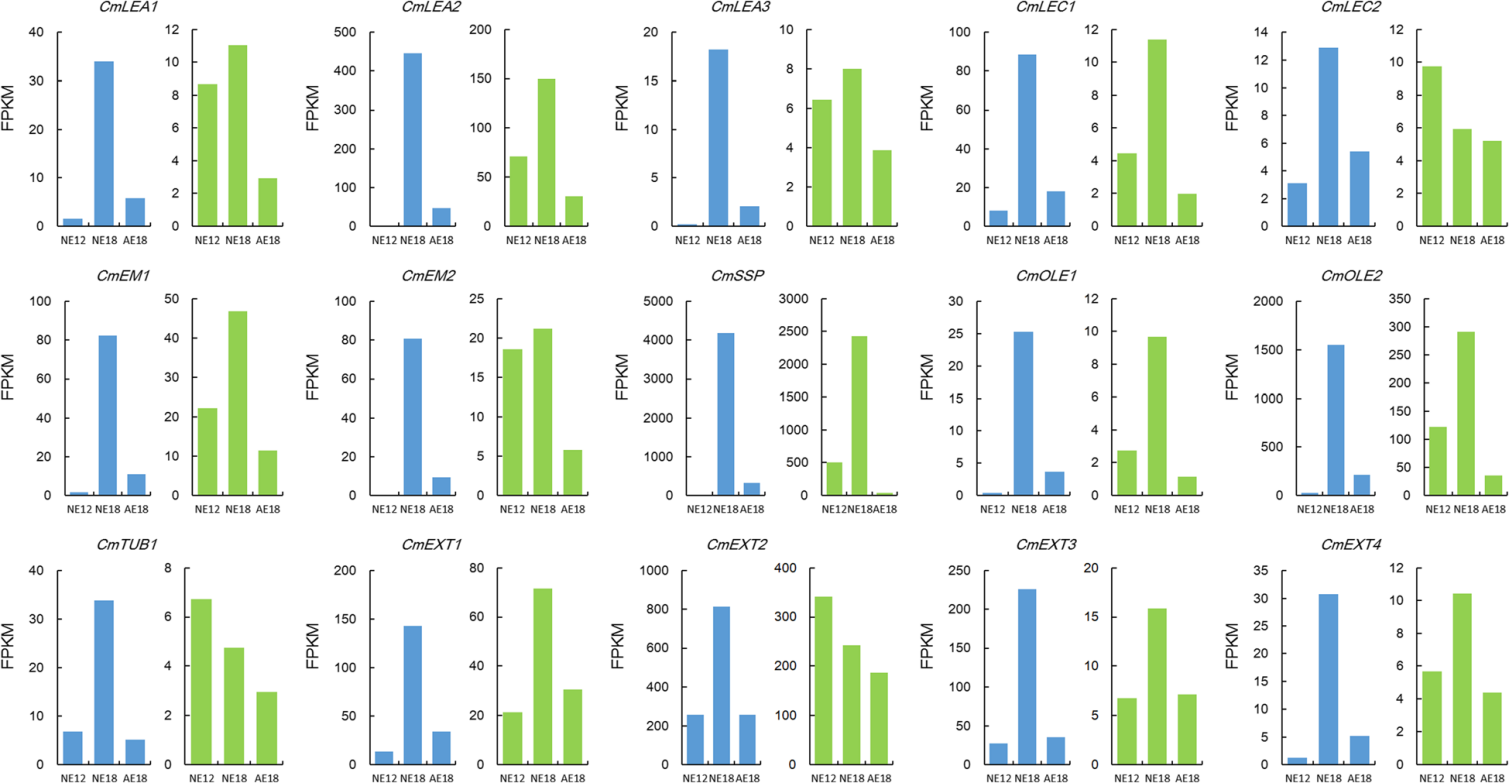
The expression patterns of DEGs in two transcriptome libraries. Blue columns represent the cross *C. morifolium* × tetraploid *C. nankingense*, and green columns represent the cross *C. morifolium* × diploid *C. nankingense*

**Fig. 4:**
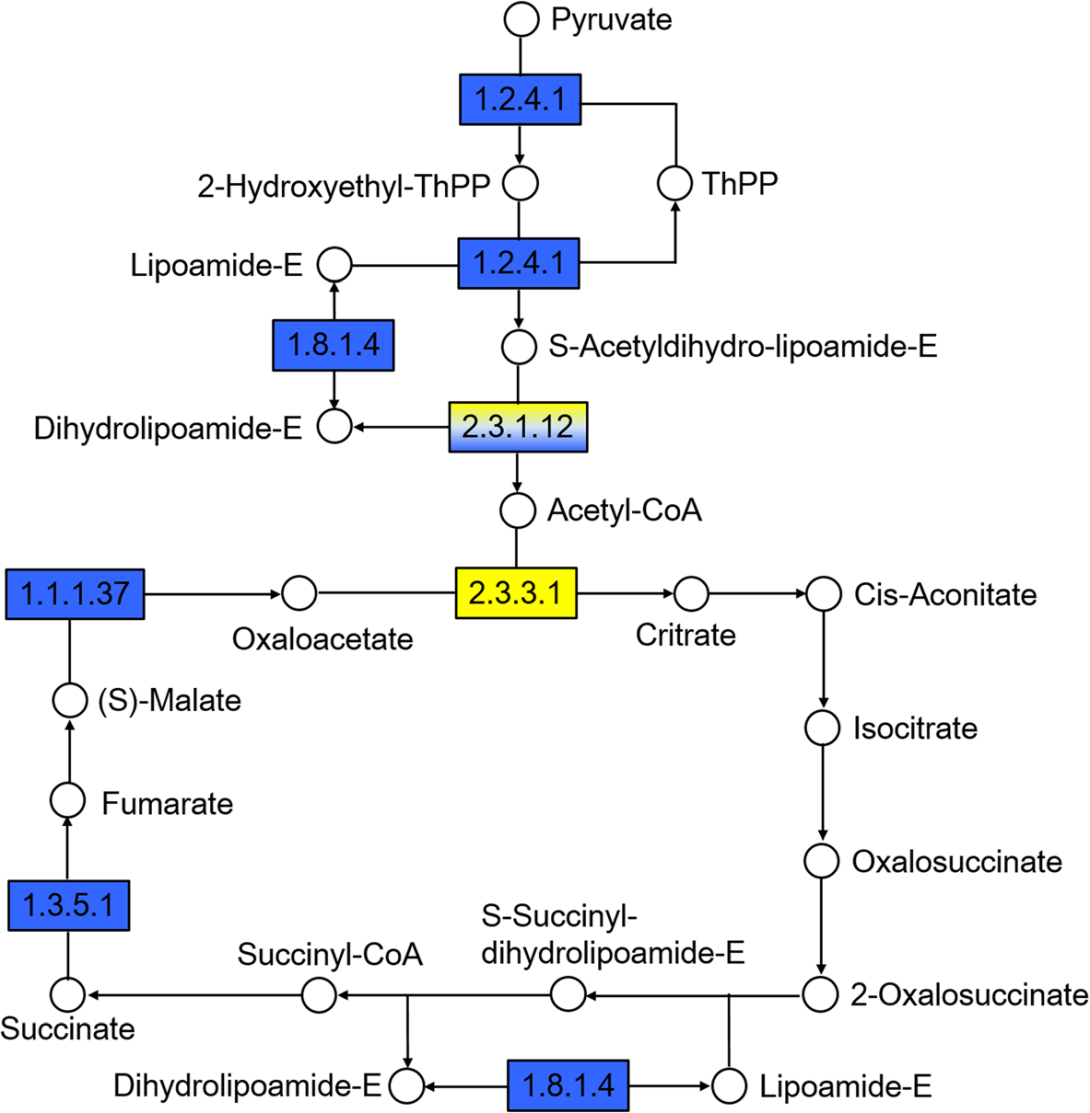
Analysis of the tricarboxylic acid (TCA) cycle pathway in normal and abnormal embryos 18 DAP in the cross *C. morifolium* × tetraploid *C. nankingense*. The map displays selected steps from the KEGG pathway ko00020 ‘Citrate cycle (TCA cycle)’. Yellow indicates higher relative levels and blue indicates lower levels in AE18. Enzymes are given as EC numbers: 1.2.4.1, pyruvate dehydrogenase; 1.8.1.4, dihydrolipoamide dehydrogenase; 2.3.1.12, dihydrolipoamide acetyltransferase; 2.3.3.1, citrate synthase; 1.3.5.1, succinate dehydrogenase; and 1.1.1.37, malate dehydrogenase

**Fig. 5:**
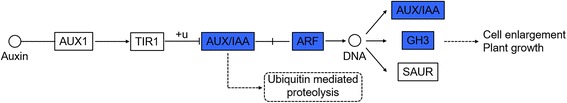
Analysis of the pathway related to auxin signal transduction in normal and abnormal embryos 18 DAP in the cross *C. morifolium* × tetraploid *C. nankingense*. The map displays selected steps from the KEGG pathway ko04075 ‘Plant hormone signal transduction’. Blue indicates the lower expression level of genes in AE18

**Fig. 6:**
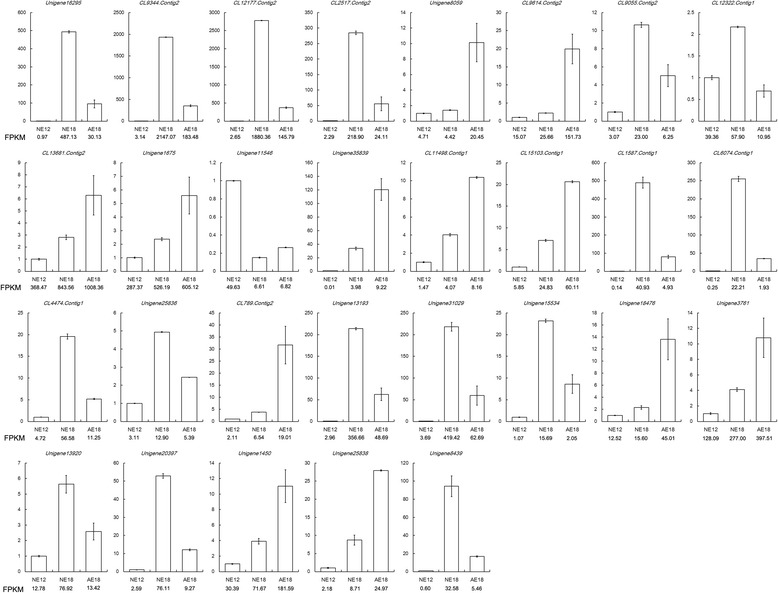
Validation of the RNA-Seq results by qRT-PCR. FPKM represents the gene abundance in the sequencing data of the transcriptome libraries
