# Supplementary figures and images for: Chromosome doubling to overcome the chrysanthemum cross barrier based on insight from transcriptomic and proteomic analyses
Source: BMC Genomics. 2016 Aug 9;17:585. doi: 10.1186/s12864-016-2939-0 (PMC4979184; doi:10.1186/s12864-016-2939-0)

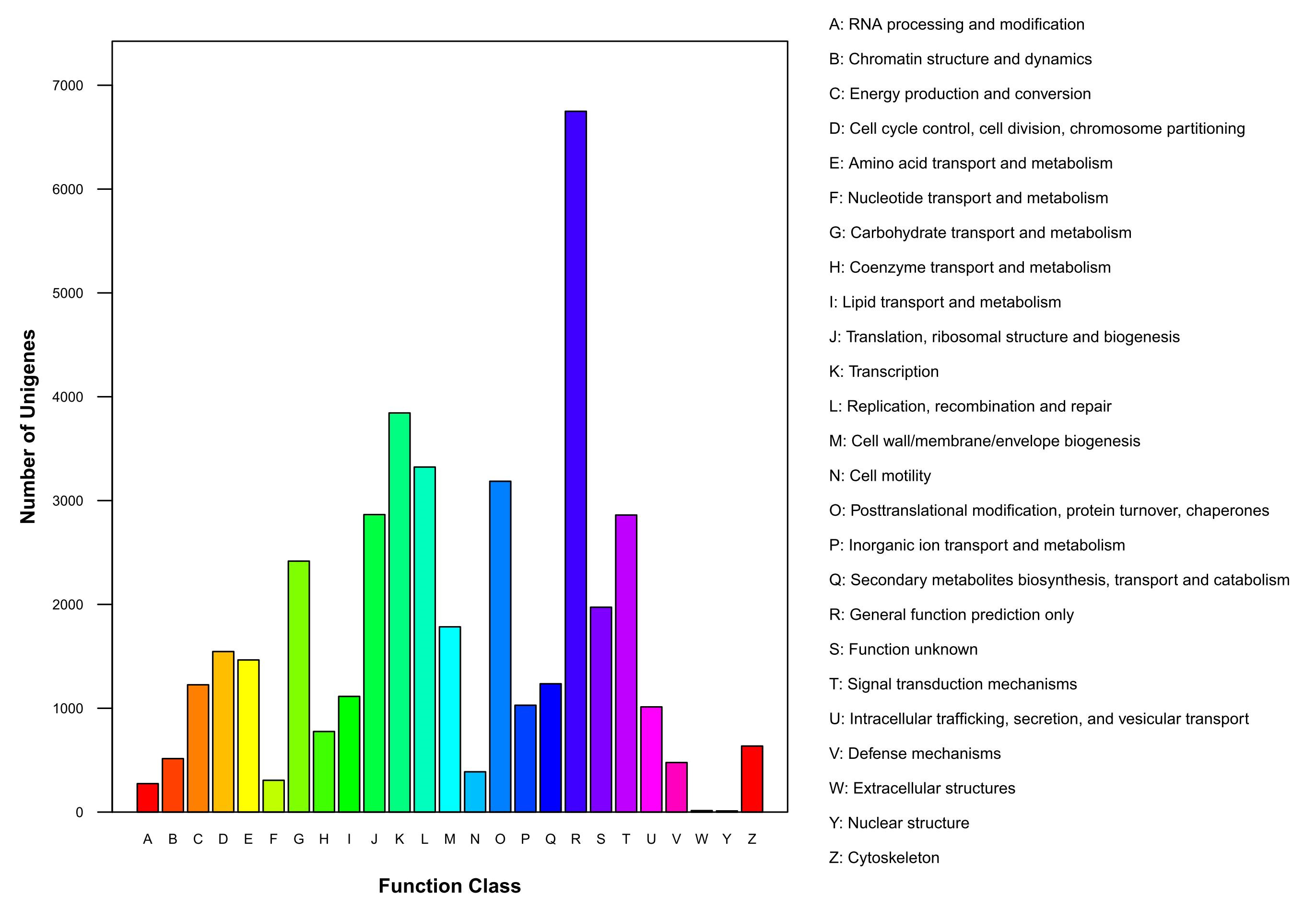

Supplement: Additional file 1: Figure S1. — Clusters of orthologous group (COG) function classification of the embryo transcriptome. The 20,391 sequences were grouped into 25 categories. (TIF 1297 kb) [file 12864_2016_2939_MOESM1_ESM.tif]

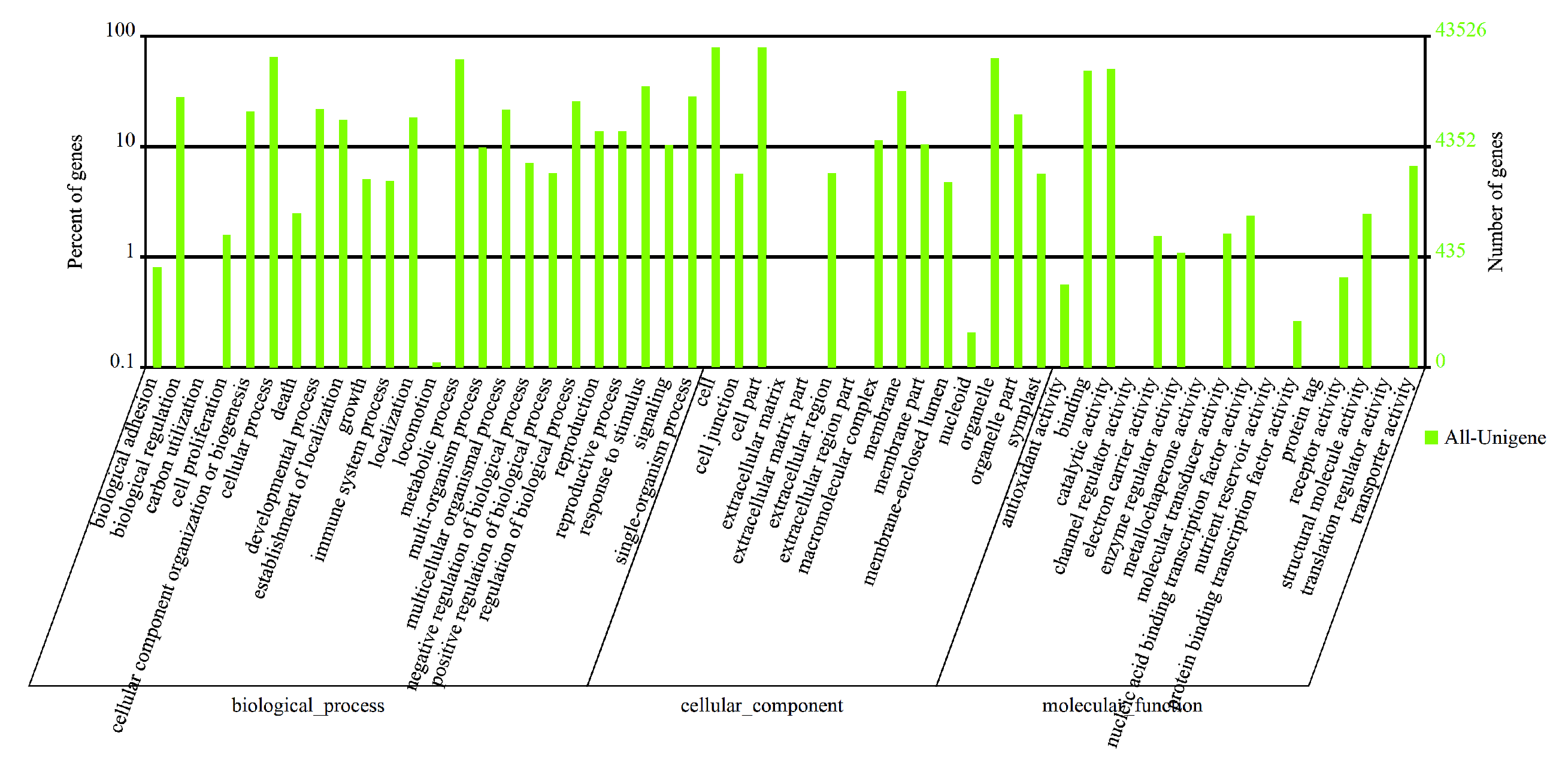

Supplement: Additional file 2: Figure S2. — Gene Ontology (GO) classification of the embryo transcriptome. The transcriptome datasets were grouped into three GO classifications: biological process, cellular component and molecular function. (TIF 380 kb) [file 12864_2016_2939_MOESM2_ESM.tif]

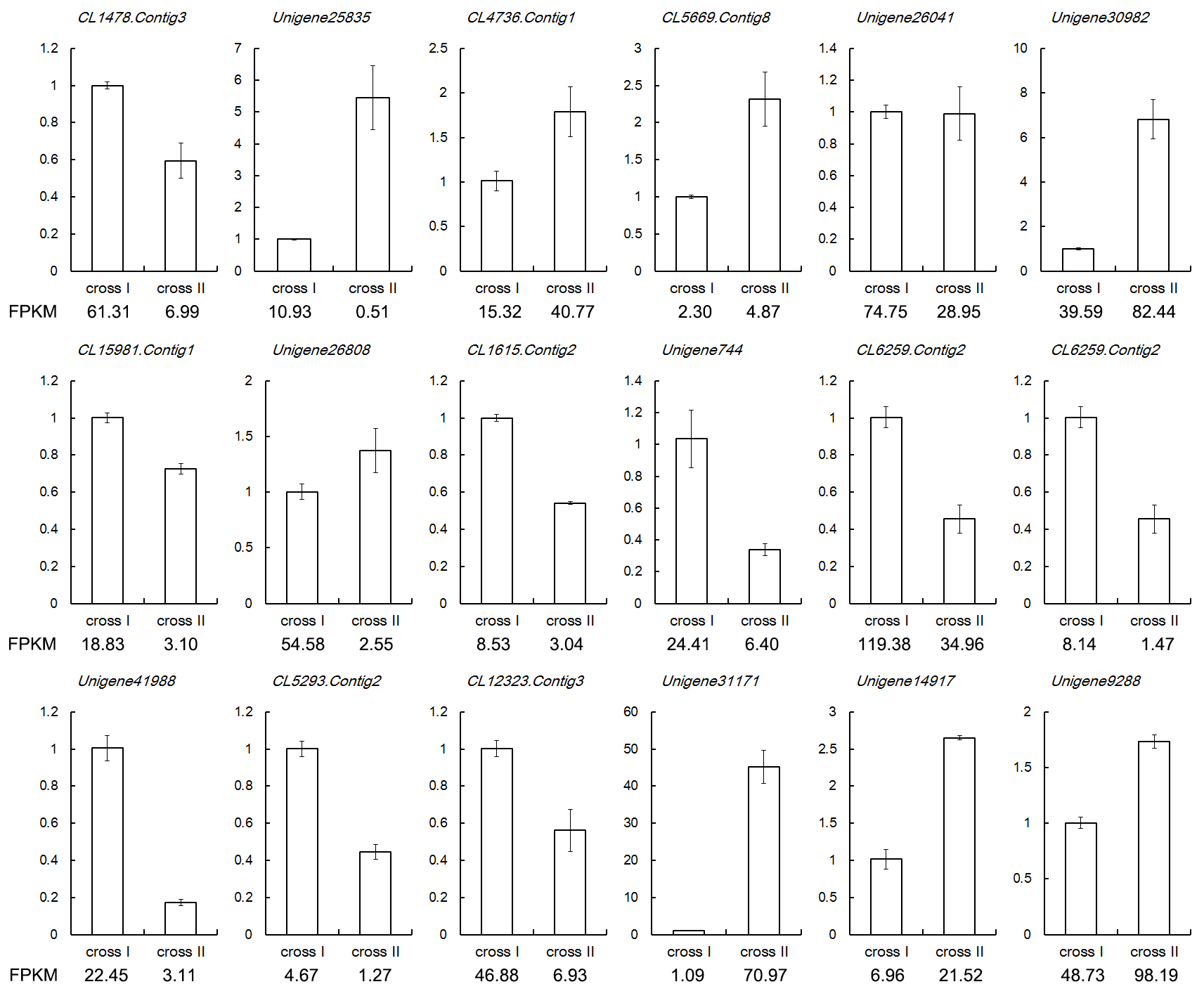

Supplement: Additional file 5: Figure S3. — qRT-PCR validation of the DEGs involved in embryo development at 12 DAP in two crosses. (TIF 165 kb) [file 12864_2016_2939_MOESM5_ESM.tif]

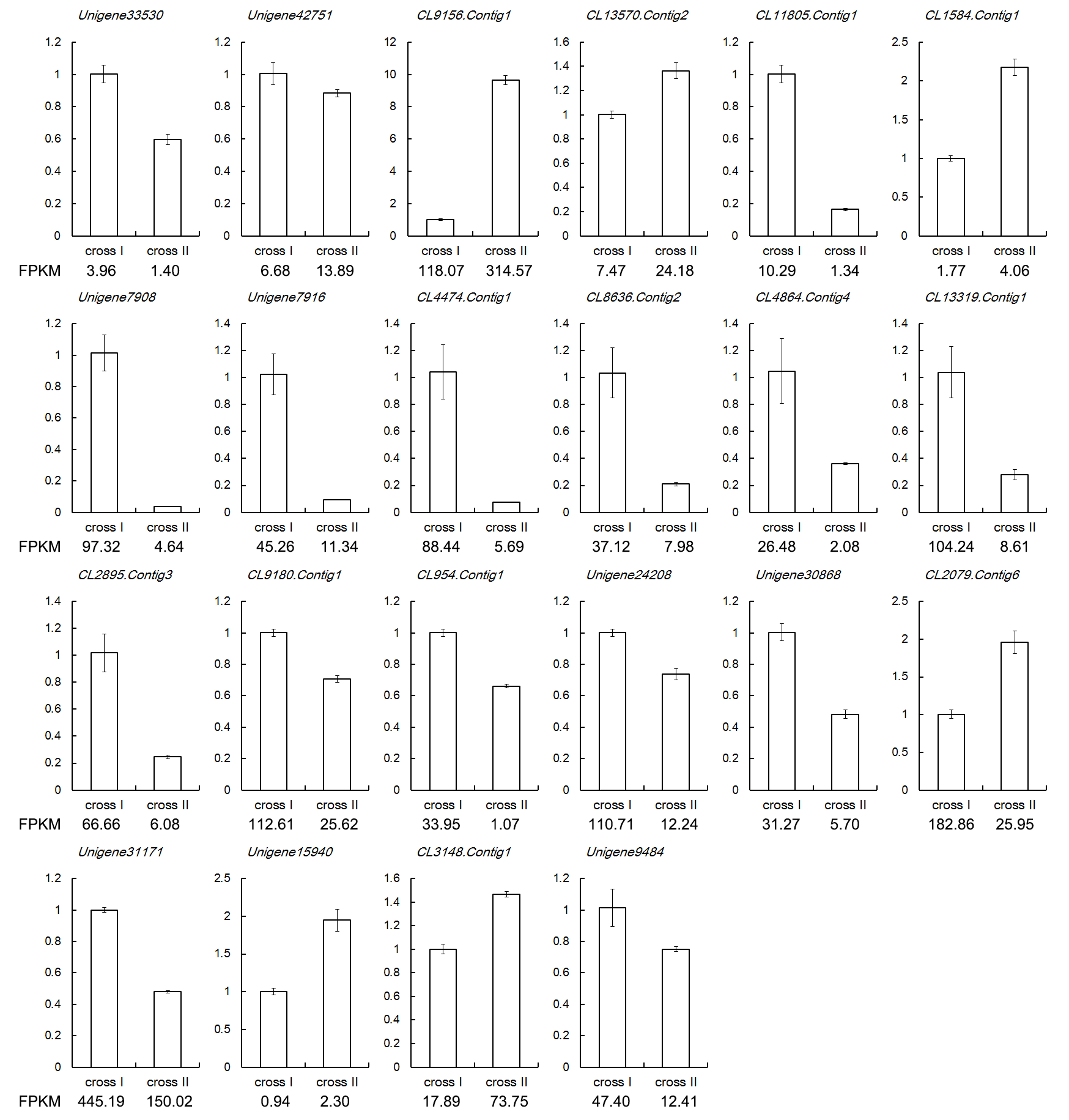

Supplement: Additional file 6. Figure S4. — qRT-PCR validation of the DEGs involved in embryo development at 18 DAP in two crosses. (TIF 204 kb) [file 12864_2016_2939_MOESM6_ESM.tif]
