# Supplementary material for: Chromosome doubling to overcome the chrysanthemum cross barrier based on insight from transcriptomic and proteomic analyses
Source: BMC Genomics. 2016 Aug 9;17:585. doi: 10.1186/s12864-016-2939-0 (PMC4979184; doi:10.1186/s12864-016-2939-0)
Supplement: Additional file 3: Table S1. — The 128 KEGG pathways. (DOCX 22 kb) [file 12864_2016_2939_MOESM3_ESM.docx]

Table S1. The 128 KEGG pathways.

| Number | Pathway | All genes with pathway annotation (33594) | Pathway ID |
| --- | --- | --- | --- |
| 1 | Metabolic pathways | 7617 (22.67%) | ko01100 |
| 2 | Biosynthesis of secondary metabolites | 3786 (11.27%) | ko01110 |
| 3 | Plant-pathogen interaction | 1905 (5.67%) | ko04626 |
| 4 | Plant hormone signal transduction | 1668 (4.97%) | ko04075 |
| 5 | RNA transport | 1287 (3.83%) | ko03013 |
| 6 | Spliceosome | 1250 (3.72%) | ko03040 |
| 7 | Endocytosis | 1171 (3.49%) | ko04144 |
| 8 | Protein processing in endoplasmic reticulum | 1042 (3.1%) | ko04141 |
| 9 | Glycerophospholipid metabolism | 993 (2.96%) | ko00564 |
| 10 | Ribosome | 914 (2.72%) | ko03010 |
| 11 | Starch and sucrose metabolism | 882 (2.63%) | ko00500 |
| 12 | Purine metabolism | 790 (2.35%) | ko00230 |
| 13 | mRNA surveillance pathway | 771 (2.3%) | ko03015 |
| 14 | Ether lipid metabolism | 747 (2.22%) | ko00565 |
| 15 | Ribosome biogenesis in eukaryotes | 732 (2.18%) | ko03008 |
| 16 | Pyrimidine metabolism | 709 (2.11%) | ko00240 |
| 17 | RNA degradation | 686 (2.04%) | ko03018 |
| 18 | Ubiquitin mediated proteolysis | 673 (2%) | ko04120 |
| 19 | Phenylpropanoid biosynthesis | 592 (1.76%) | ko00940 |
| 20 | Glycolysis / Gluconeogenesis | 465 (1.38%) | ko00010 |
| 21 | Oxidative phosphorylation | 444 (1.32%) | ko00190 |
| 22 | RNA polymerase | 423 (1.26%) | ko03020 |
| 23 | Pentose and glucuronate interconversions | 420 (1.25%) | ko00040 |
| 24 | Stilbenoid, diarylheptanoid and gingerol biosynthesis | 412 (1.23%) | ko00945 |
| 25 | ABC transporters | 401 (1.19%) | ko02010 |
| 26 | Amino sugar and nucleotide sugar metabolism | 398 (1.18%) | ko00520 |
| 27 | Aminoacyl-tRNA biosynthesis | 385 (1.15%) | ko00970 |
| 28 | Pyruvate metabolism | 379 (1.13%) | ko00620 |
| 29 | Flavonoid biosynthesis | 358 (1.07%) | ko00941 |
| 30 | Phagosome | 354 (1.05%) | ko04145 |
| 31 | Nucleotide excision repair | 338 (1.01%) | ko03420 |
| 32 | Limonene and pinene degradation | 320 (0.95%) | ko00903 |
| 33 | Circadian rhythm - plant | 320 (0.95%) | ko04712 |
| 34 | Peroxisome | 309 (0.92%) | ko04146 |
| 35 | Homologous recombination | 300 (0.89%) | ko03440 |
| 36 | Zeatin biosynthesis | 293 (0.87%) | ko00908 |
| 37 | DNA replication | 287 (0.85%) | ko03030 |
| 38 | Glycine, serine and threonine metabolism | 274 (0.82%) | ko00260 |
| 39 | Phosphatidylinositol signaling system | 262 (0.78%) | ko04070 |
| 40 | Terpenoid backbone biosynthesis | 257 (0.77%) | ko00900 |
| 41 | Carbon fixation in photosynthetic organisms | 254 (0.76%) | ko00710 |
| 42 | Mismatch repair | 252 (0.75%) | ko03430 |
| 43 | Cyanoamino acid metabolism | 243 (0.72%) | ko00460 |
| 44 | Galactose metabolism | 239 (0.71%) | ko00052 |
| 45 | Fatty acid metabolism | 238 (0.71%) | ko00071 |
| 46 | Arginine and proline metabolism | 234 (0.7%) | ko00330 |
| 47 | Cysteine and methionine metabolism | 232 (0.69%) | ko00270 |
| 48 | Phenylalanine metabolism | 229 (0.68%) | ko00360 |
| 49 | Glutathione metabolism | 227 (0.68%) | ko00480 |
| 50 | Flavone and flavonol biosynthesis | 227 (0.68%) | ko00944 |
| 51 | Other glycan degradation | 220 (0.65%) | ko00511 |
| 52 | Inositol phosphate metabolism | 214 (0.64%) | ko00562 |
| 53 | Ascorbate and aldarate metabolism | 213 (0.63%) | ko00053 |
| 54 | Carotenoid biosynthesis | 206 (0.61%) | ko00906 |
| 55 | Glycerolipid metabolism | 202 (0.6%) | ko00561 |
| 56 | Basal transcription factors | 201 (0.6%) | ko03022 |
| 57 | Fructose and mannose metabolism | 201 (0.6%) | ko00051 |
| 58 | Citrate cycle (TCA cycle) | 198 (0.59%) | ko00020 |
| 59 | Regulation of autophagy | 191 (0.57%) | ko04140 |
| 60 | Tyrosine metabolism | 184 (0.55%) | ko00350 |
| 61 | Valine, leucine and isoleucine degradation | 183 (0.54%) | ko00280 |
| 62 | Base excision repair | 183 (0.54%) | ko03410 |
| 63 | Glyoxylate and dicarboxylate metabolism | 180 (0.54%) | ko00630 |
| 64 | Propanoate metabolism | 179 (0.53%) | ko00640 |
| 65 | Phenylalanine, tyrosine and tryptophan biosynthesis | 176 (0.52%) | ko00400 |
| 66 | Proteasome | 175 (0.52%) | ko03050 |
| 67 | alpha-Linolenic acid metabolism | 174 (0.52%) | ko00592 |
| 68 | Protein export | 151 (0.45%) | ko03060 |
| 69 | Cutin, suberine and wax biosynthesis | 151 (0.45%) | ko00073 |
| 70 | Pentose phosphate pathway | 149 (0.44%) | ko00030 |
| 71 | Porphyrin and chlorophyll metabolism | 147 (0.44%) | ko00860 |
| 72 | Diterpenoid biosynthesis | 144 (0.43%) | ko00904 |
| 73 | Glycosylphosphatidylinositol(GPI)-anchor biosynthesis | 143 (0.43%) | ko00563 |
| 74 | Tryptophan metabolism | 140 (0.42%) | ko00380 |
| 75 | beta-Alanine metabolism | 135 (0.4%) | ko00410 |
| 76 | Biosynthesis of unsaturated fatty acids | 134 (0.4%) | ko01040 |
| 77 | Sesquiterpenoid and triterpenoid biosynthesis | 134 (0.4%) | ko00909 |
| 78 | Alanine, aspartate and glutamate metabolism | 133 (0.4%) | ko00250 |
| 79 | Fatty acid biosynthesis | 131 (0.39%) | ko00061 |
| 80 | Sphingolipid metabolism | 131 (0.39%) | ko00600 |
| 81 | N-Glycan biosynthesis | 127 (0.38%) | ko00510 |
| 82 | Steroid biosynthesis | 126 (0.38%) | ko00100 |
| 83 | Lysine degradation | 123 (0.37%) | ko00310 |
| 84 | Ubiquinone and other terpenoid-quinone biosynthesis | 123 (0.37%) | ko00130 |
| 85 | SNARE interactions in vesicular transport | 121 (0.36%) | ko04130 |
| 86 | Glycosaminoglycan degradation | 118 (0.35%) | ko00531 |
| 87 | Natural killer cell mediated cytotoxicity | 113 (0.34%) | ko04650 |
| 88 | Nitrogen metabolism | 109 (0.32%) | ko00910 |
| 89 | Isoflavonoid biosynthesis | 103 (0.31%) | ko00943 |
| 90 | Butanoate metabolism | 99 (0.29%) | ko00650 |
| 91 | Pantothenate and CoA biosynthesis | 98 (0.29%) | ko00770 |
| 92 | Photosynthesis | 95 (0.28%) | ko00195 |
| 93 | Valine, leucine and isoleucine biosynthesis | 93 (0.28%) | ko00290 |
| 94 | Circadian rhythm - mammal | 91 (0.27%) | ko04710 |
| 95 | Isoquinoline alkaloid biosynthesis | 87 (0.26%) | ko00950 |
| 96 | Brassinosteroid biosynthesis | 84 (0.25%) | ko00905 |
| 97 | Glycosphingolipid biosynthesis - ganglio series | 81 (0.24%) | ko00604 |
| 98 | Benzoxazinoid biosynthesis | 81 (0.24%) | ko00402 |
| 99 | Fatty acid elongation | 76 (0.23%) | ko00062 |
| 100 | Sulfur metabolism | 75 (0.22%) | ko00920 |
| 101 | Tropane, piperidine and pyridine alkaloid biosynthesis | 75 (0.22%) | ko00960 |
| 102 | Linoleic acid metabolism | 64 (0.19%) | ko00591 |
| 103 | Histidine metabolism | 64 (0.19%) | ko00340 |
| 104 | Selenocompound metabolism | 61 (0.18%) | ko00450 |
| 105 | Arachidonic acid metabolism | 58 (0.17%) | ko00590 |
| 106 | Lysine biosynthesis | 55 (0.16%) | ko00300 |
| 107 | Nicotinate and nicotinamide metabolism | 50 (0.15%) | ko00760 |
| 108 | Riboflavin metabolism | 46 (0.14%) | ko00740 |
| 109 | Monoterpenoid biosynthesis | 46 (0.14%) | ko00902 |
| 110 | One carbon pool by folate | 45 (0.13%) | ko00670 |
| 111 | Non-homologous end-joining | 42 (0.13%) | ko03450 |
| 112 | Photosynthesis - antenna proteins | 41 (0.12%) | ko00196 |
| 113 | Glucosinolate biosynthesis | 39 (0.12%) | ko00966 |
| 114 | Vitamin B6 metabolism | 38 (0.11%) | ko00750 |
| 115 | Sulfur relay system | 37 (0.11%) | ko04122 |
| 116 | Folate biosynthesis | 35 (0.1%) | ko00790 |
| 117 | Synthesis and degradation of ketone bodies | 35 (0.1%) | ko00072 |
| 118 | Other types of O-glycan biosynthesis | 31 (0.09%) | ko00514 |
| 119 | Glycosphingolipid biosynthesis - globo series | 26 (0.08%) | ko00603 |
| 120 | Anthocyanin biosynthesis | 26 (0.08%) | ko00942 |
| 121 | Thiamine metabolism | 23 (0.07%) | ko00730 |
| 122 | Taurine and hypotaurine metabolism | 22 (0.07%) | ko00430 |
| 123 | C5-Branched dibasic acid metabolism | 22 (0.07%) | ko00660 |
| 124 | Indole alkaloid biosynthesis | 19 (0.06%) | ko00901 |
| 125 | Lipoic acid metabolism | 11 (0.03%) | ko00785 |
| 126 | Biotin metabolism | 8 (0.02%) | ko00780 |
| 127 | Caffeine metabolism | 3 (0.01%) | ko00232 |
| 128 | Betalain biosynthesis | 2 (0.01%) | ko00965 |
