# Supplementary material for: Chromosome doubling to overcome the chrysanthemum cross barrier based on insight from transcriptomic and proteomic analyses
Source: BMC Genomics. 2016 Aug 9;17:585. doi: 10.1186/s12864-016-2939-0 (PMC4979184; doi:10.1186/s12864-016-2939-0)
Supplement: Additional file 7: Table S3. — Primer sequences for qRT-PCR. (DOCX 17 kb) [file 12864_2016_2939_MOESM7_ESM.docx]

**Table S3.** Primer sequences for qRT-PCR

| Unigene ID | Forward Primer | Reverse Primer |
| --- | --- | --- |
| Unigene16295 | GACAAGGTGATCGTGGTGGT | ATCTACGTCCCTGGTGCTCT |
| CL9344.Contig2 | CTGTCTTGGCCACTACACGT | TCGAACCGTGGTCCTCAAAG |
| CL12177.Contig2 | AGCTTCGTGAGGGACAAGTG | TGAACAGGCATGCTTCGGAT |
| CL2517.Contig2 | GCTAGTGCCAGCTGTCATCA | TCTGCGCAGTGTCATCCAAT |
| Unigene8059 | TGCGTGGAAAGACATCTGCT | TTCTTGCGCTTCCAACATGC |
| CL9614.Contig2 | AAGTTGCGGGATCTCCACAG | AAACCGGTCGGCATTGAGAT |
| CL9055.Contig2 | CCGCAGTACAGTAGTGGCAA | GCCATTGCACTGGACGATTC |
| CL12322.Contig1 | ATGATCACTGCTGGCTGCTT | GCGTCCCATCTCAAAGACCA |
| CL13681.Contig2 | AAGCTGAGAACGTGTGGAGG | GCCAGTCGTAAACAGTGGGA |
| Unigene1675 | CATGAACGTGGAAGTCCCGA | GATCACTTCAGGGAACGCCA |
| Unigene11546 | CCTCCCTCTTTGAGCACAGG | TGGAGACATGCTTTGCGGAT |
| Unigene35839 | TCGCCAGAATCATCCATCGG | AAGGCGGATGTTAAGAGGCC |
| CL11498.Contig1 | ATAGAGGCGTGAGAAAGCGG | ATAAGCCAAAGCCGCCTCTT |
| CL15103.Contig1 | TCTCGGTTCTAGCTCAGCCT | ACCTTCACGCACTGAGTCAG |
| CL1587.Contig1 | GGCCGCCACCAAGGATTATA | TCGTGTCCTTGCCTTCCTTC |
| CL6074.Contig1 | AAACAAGGTGCTGGCTCAGT | TTTCGTAAGCCCCGTCCTTC |
| CL4474.Contig1 | GGCCATGAGCAAACTAGGGT | ACGTTCTCCGCCATCAAACT |
| Unigene25836 | ATCCTTCCTTCGCATGCCAA | TTGCTCACGTTGGCACCTAT |
| CL789.Contig2 | TTTTGACCCGAGAGACGTGG | CTTTCCCGTGAGACGAACCA |
| Unigene13193 | GAGACAAGTGACTGGGACGG | CCACCATGAACCTGACCCTC |
| Unigene31029 | AGGGACATTTGGGCTAACCG | TAGCCTGGCCCATTTCCTTG |
| Unigene15534 | CATCATCATCCTCGGACGCA | GTGTTATGAGGCGCATGCTG |
| Unigene18476 | CGAAAGGATGCATGGCTGTG | ACGTCGCAAGGGATGTTGAT |
| Unigene3761 | GGACCACATTGGCACCAAAC | AACTGTCACATCCCCACACC |
| Unigene13920 | TGAGGACTTGGCTGCTCTTG | CGCCATCATCATCTTCCCCA |
| Unigene20397 | ACCCATGATCACGGTGGTTC | TACGGAATCCATTACCGCGG |
| Unigene1450 | CACCAACTAACCCCTCGGAC | GAAATCGCAGCTGCTAAGGC |
| Unigene25838 | GGCGTGTGGAGTGAAGAAGA | TCCTCGAGGTGTCATGGGAT |
| Unigene8439 | TGTCCCACACACCACTTCAC | CTCCTGGTGGTGCTTGAGAG |
| EF1a | TTTGGTATCTGGTCCTGGAG | CCATTCAAGCGACAGACTCA |
| CL1478.Contig3 | TGTCTTGCCACCCTCATCAC | GGTGAGGAGAACGTGCTTGA |
| Unigene25835 | GTTTGCTGCTATAACCGCGG | CAAAACACACGCGGCTACAA |
| CL4736.Contig1 | GAAAGTCTCGGCCTGAGCTT | CCTCCCGCCCAAAAGATTCT |
| CL5669.Contig8 | GTGCTGTTAGGGGAGCCATT | TGTCTGGCCATACTGTGCAG |
| Unigene26041 | AACTCGTTCGCAGCTCTTGA | AGTAAAGTGGCGGAGTTGGG |
| Unigene30982 | CTGCAGAAGACGGTGTCTGT | GAAACAGGTTGGTGCGTGAC |
| CL15981.Contig1 | GTATCCGTCTCAGAGCCAGC | CAGAAGGCGGCACTTGACTA |
| Unigene26808 | CATGCAGTGTGGCGATCAAG | AACCACCCTCAGTTGCACAA |
| CL1615.Contig2 | CTTGCACTCAAGGGCAAAGC | TCGAATCAGGGAGGTACGGA |
| Unigene744 | GTGGATGGTCACACTCCAGG | GTCTTCTGGGGTTTGGGGAG |
| CL6259.Contig2 | CGAGTCAAAAGCCGATGCAG | GTGACCACCCTTACGGATGG |
| Unigene41988 | GCGTATATGAGGTTGGCGGA | CCCACACCCATAAACCGACA |
| CL5293.Contig2 | TTGCTGCAGTTCCATGTTGC | CCTGAACGTTGTGTAGCCCT |
| CL16042.Contig2 | CCAACACACTGAGCTGCAAC | GGGAGCGGGTAGGTAAACTT |
| CL12323.Contig3 | AGGAAGCAATTCAGGAGGCC | GCGTCATTACCGACCAATGC |
| Unigene31171 | CAAGCTAAGGGACAAGCCGA | ATAGCTTCCGGTCTGGTCCT |
| Unigene14917 | AGCCAAGCCGATTTAGACCC | TCGGTTTTGGGTTCGGGTAG |
| Unigene9288 | CAAGGTGGATGGTGGTACCC | GAGTGCCACGTGTTGTAGGA |
| Unigene33530 | AAATGATCTGGGTGGGCTGG | GTGCAGCTACAGCCTTACCA |
| Unigene42751 | AAGCTCACAGTACCGTGGTG | CCTGCTTCCCACAATCCCAT |
| CL9156.Contig1 | GCTGCGGCTAGAGCTTATGA | CGGTTTCGTCTCCTTCACCA |
| CL13570.Contig2 | AGGGGAGTTGAAGCAGTTGG | GTTGGTAACCACCCTAGGGC |
| CL11805.Contig1 | GGGCTCGCGTATCTTCATCA | CAGCTACTCGCGACATAGCA |
| CL1584.Contig1 | CGATATGAGGCTCGCTGTGT | CAATTCTGGTGGGCCCTCTT |
| Unigene7908 | TCTACCCCCTGGTGGATCAG | TTAGCTGACGACCGAAACCC |
| Unigene7916 | ATAGGCGTCAACTATGGCCG | TCACCCGCTGCAGACTTTAG |
| CL4474.Contig1 | GGCCATGAGCAAACTAGGGT | ACGTTCTCCGCCATCAAACT |
| CL8636.Contig2 | ACCTCCTCCTCCTCATCCAC | GGAGTGATTGAGGGTGCGAA |
| CL4864.Contig4 | ACAGAGGTGAAGAGCCATGC | CAGACACACATGGACCGTGA |
| CL13319.Contig1 | ACAGCAGCAGCAAGACATCT | ACCGTACAGTGCTCCAACTG |
| CL2895.Contig3 | TGTCGGAACTGATTCGCACA | CCACACCAGGCAAAACCATG |
| CL9180.Contig1 | ATGGCACTGTGACACGTCAT | CCTCGGGTCCAAGCAAAGAT |
| CL954.Contig1 | GACCAGGCCGAAGAGATAGC | GCTGACAACTTTGCCTTGGG |
| Unigene24208 | TCAAAAACTGCGGCCCAATG | GCCGTTACACCCGTCGATAT |
| Unigene30868 | GATCGGCTGTTTCGTTACGC | ATTCGTTCACGGTCACCTCC |
| CL2079.Contig6 | ACCATCACCATCGTCAACCC | TGATGTCAGCAGCATCACCA |
| Unigene31171 | CAAGCTAAGGGACAAGCCGA | ATAGCTTCCGGTCTGGTCCT |
| Unigene15940 | TGTTGACCCAAGGCTATCGG | GCAACACAGGACATTGCGAG |
| CL3148.Contig1 | CAGGAATGGTGGTGCAGCTA | TCCCCTGATGTCGAGTTCCT |
| Unigene9484 | ACTGCTCTGATTCAGGTGGC | AGCTAAGACGGCTGTCCCTA |
